# Sleep patterns and risks of incident cardiovascular disease and mortality among people with type 2 diabetes: a prospective study of the UK Biobank

**DOI:** 10.1186/s13098-024-01261-8

**Published:** 2024-01-11

**Authors:** Jinxia Hu, Xuanyang Wang, Licheng Cheng, Keke Dang, Zhu Ming, Xinmiao Tao, Xiaoqing Xu, Shuvan Kumar Sarker, Ying Li

**Affiliations:** Department of Nutrition and Food Hygiene, Key Laboratory of Precision Nutrition and Health, School of Public Health, Ministry of Education, Harbin Medical University, 157 Baojian Road, Heilongjiang, 150081 People’s Republic of China

**Keywords:** Cardiovascular disease, Type 2 diabetes mellitus (T2DM), Sleep pattern, Polygenic risk score (PRS)

## Abstract

**Background:**

To explore the relationship between sleep patterns and cardiovascular disease (CVD) incidence and mortality risk in a population with type 2 diabetes through a UK Biobank sample.

**Methods:**

A total of 6860 patients with type 2 diabetes were included in this study. Five sleep factors (including Chronotype, sleep duration, insomnia, daytime sleepiness, and snoring) were collected as a questionnaire. The calculation generates a sleep score of 0–5, and then three sleep patterns were defined based on the sleep scores: poor sleep pattern (0–2), Intermediate sleep pattern (3–4), and healthy sleep pattern (5). HRs and 95% confidence intervals were calculated by multivariate COX proportional risk model adjustment. Restricted cubic splines were used to validate linear associations between sleep scores CVD events.

**Results:**

Our results found a reduced risk of CVD events in individuals with healthy sleep patterns compared to participants with poor sleep patterns. CVD Mortality (HR, 0.690; 95% CI 0.519–0.916), ASCVD (Atherosclerosis CVD) (HR, 0.784; 95% CI 0.671–0.915), CAD (Coronary Artery Disease) (HR, 0.737; 95% CI 0.618–0.879), PAD (Peripheral Arterial Disease) (HR, 0.612; 95% CI 0.418–0.896), Heart Failure (HR, 0.653; 95% CI 0.488–0.875). Restricted cubic spline responded to a negative linear correlation between sleep scores and CVD Mortality, ASCVD, CAD, PAD, and Heart Failure.

**Conclusions:**

Healthy sleep patterns are significantly associated with a reduced risk of CVD Mortality, ASCVD, CAD, PAD, and Heart Failure in the diabetes population.

**Supplementary Information:**

The online version contains supplementary material available at 10.1186/s13098-024-01261-8.

## Introduction

Diabetes mellitus is a chronic metabolic disorder that leads to high blood glucose levels. As the disease progresses, it impairs physical functions such as the heart, kidneys, blood vessels, and nerves. It also affects psychological and social functioning, reducing the quality of life and increasing disability and mortality rates [1–3]. Unfortunately, type 2 diabetes is a chronic and incurable disease [4]. Cardiovascular disease (CVD) is the leading cause of death in patients with type 2 diabetes. A combination of diabetes mellitus and CVD is multifactorial, and controlling cardiovascular risk factors can significantly reduce the incidence of cardiovascular events [5–7]. The 2015 joint scientific statement by the American Heart Association (AHA) and American Diabetes Association (ADA) titled “Update on Prevention of Cardiovascular Disease in Adults with Type 2 Diabetes” highlights the importance of modifying various risk factors that contribute to CVD in people with diabetes. Apart from traditional lifestyle behaviors, there is mounting evidence that unhealthy sleep habits significantly increase the risk of CVD [8–10]. This is particularly true for individuals with type 2 diabetes [11]. For example, long or short time of sleep [12–18], late chronotype [19, 20], insomnia [21–26], snoring [27, 28], and daytime sleepiness [29, 30] can increase the risk of CVD by 10–30%. However, studies have shown that these sleep behaviors are often interrelated, and combinations of different sleep behaviors have the potential to be partially cumulative in health outcomes. Therefore, the combined effect of different sleep behaviors may have a cumulative impact on overall health outcomes. The study found that the combination of insomnia symptoms and long sleep duration increased the impact of a single sleep factor on the risk of acute myocardial infarction by nearly 20 [31]. Several prospective cohort studies conducted in the United States suggest that insomnia and a lack of sleep can increase the risk of cardiovascular events [32]. Additionally, a study that included participants from 21 countries found that people who take long daytime naps may reduce the risk of CVD in those who experience sleep deprivation at night [33]. Based on the complexity and relevance of various sleep behaviors, Fan et al. devised a sleep score by combining five sleep factors (chronotype, sleep duration, insomnia, snoring, and daytime sleepiness). They found a significant negative correlation between sleep score and CVD, highlighting the importance of maintaining good sleep habits [34]. The validity of the result has been confirmed by various population studies. However, most current studies only focus on the relationship between single sleep behaviors and the occurrence of CVD events or on the relationship between sleep score characteristics and specific CVD diseases. The relationship between sleep patterns and CVD risk, particularly in individuals with type 2 diabetes, is not well understood.

Therefore, in this study, we used sleep scores generated by five sleep factors as the primary features and defined three sleep patterns (poor sleep pattern, Intermediate sleep pattern, and healthy sleep pattern). Patients with type 2 diabetes were studied to examine the association between three different sleep patterns and the occurrence of cardiovascular disease (CVD) events, including CVD deaths.

## Methods

### Study population

The UK Biobank is a national cohort study that prospectively followed over 500,000 adults aged between 40 and 69 years. The participants were recruited between 2006 and 2010 from 22 centers across the UK (A map of the 22 centres can be found here: https://www.ukbiobank.ac.uk/enable-your-research/about-our-data/baseline-assessment). We have included the follow-up data of the participants from the baseline until February 2022. This study collected extensive phenotypic and genotypic data from its participants, which includes information from questionnaires, physical measures, biological samples, and genome-wide genotyping. The participants provided information on their sleep patterns and other health-related aspects through touch-screen questionnaires and physical measurements. Blood samples were also collected for genotyping purposes. The UK Biobank research has been approved by the North West Multicenter Research Ethical Committee, and all participants provided written informed consent for the study [35].

**Fig. 1:**
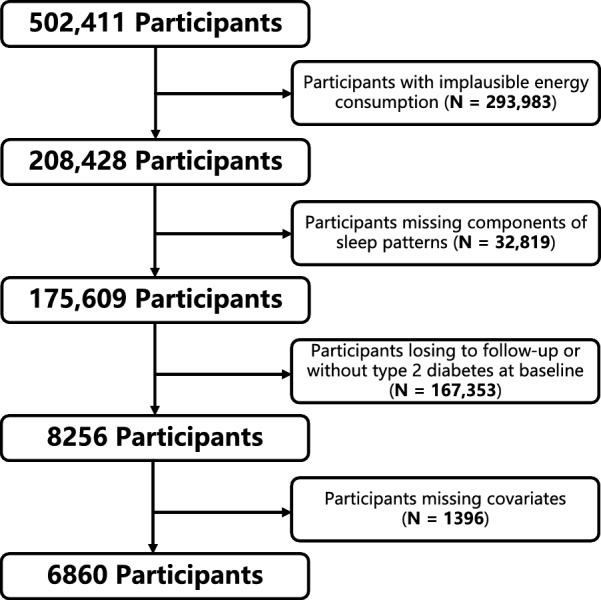
Flow chart. people with type 2 diabetes

As shown in Fig. 1, we excluded participants with missing dietary energy (n = 293,983), participants with missing sleep patterns (n = 32,819), participants with missing visits, and no type 2 diabetes at baseline (n = 167,353). Finally, we excluded participants with missing other covariates [including ethnicity (n = 30), BMI (n = 36), smoking (n = 29), drinking (n = 5), exercise (n = 1237), education (n = 28), Thompson Deprivation Index (n = 7) and participants with missing survival time (n = 24)]. We included 6860 participants with type 2 diabetes at baseline who were identified through self-reported medical history and use of anti-diabetic medication, hospital inpatient records (ICD-9 codes 250.00, 250.10, 250.20, and 250.90 and ICD-10 code E11), and abnormal glucose levels (random glucose ≥ 199.8 mg/dL or glycated hemoglobin [HbA1c] ≥ 6.5%) [36].

### Definition of sleep patterns

The sleep traits of UK Biobank participants were recorded through self-reporting in a touch screen questionnaire from 2006 to 2010. Five sleep factors, including chronotype, sleep duration, insomnia, snoring, and excessive daytime sleepiness, were defined by a specific question for each. The details of these five sleep factors have been reported previously [34] (see Additional file 1: Table S1). Low-risk sleep factors were identified as follows: (1) early-arising chronotype; (2) 7–9 h of sleep per day; ((3) no or rare occurrence of insomnia; (4) absence of snoring; and (5) low frequency of daytime sleepiness [34, 37]. We used the criteria of Fan et al. to define each sleep factor as a dichotomous variable, where “1” represented low-risk sleep factors and “0” represented high-risk sleep factors. This allowed us to generate a final sleep score ranging from 0 to 5. A score of 0–2 indicated a poor sleep pattern, 3–4 indicated an Intermediate sleep pattern and a score of 5 indicated a healthy sleep pattern.

### Definition of polygenic risk score

The genotyping process and arrays used in the UK Biobank study have been described in other articles [38]. In the current study, we selected 348 SNPs related to chronotype [39], 112 SNPs related to sleep duration [40], 248 SNPs related to insomnia [41], 41 SNPs linked to snoring [42], and 124 SNPs associated with daytime sleepiness [43] (see Additional file 2 for detailed information). Based on the selected SNPs, genetic risk scores were calculated individually for each sleep factor. Each SNP was coded as 0, 1, or 2 depending on the number of risk alleles. We constructed an equation to calculate each participant’s weighted polygenic risk score (PRS) by multiplying the risk alleles by the corresponding effect size for each SNP. PRS = β1 × SNP1 + β2 × SNP2 + … + βn × SNPn. The effect sizes (b-factors) for each SNP were obtained from the GWAS data. We categorized these individuals into three groups based on tertiles: “high genetic risk” (third quartile), “intermediate genetic risk” (second quartile), and “low genetic risk” (first quartile).

### Definition of CVD outcomes

In the UK Biobank study, we gathered information about each participant’s survival status by utilizing hospital admission data and records from death registries. We obtained the hospital admission and diagnostic details through linked patient data, specifically the Health of England and Wales dataset and the Scottish Morbidity Record. Detailed information is available online for those interested in learning more about the linkage program https://biobank.ctsu.ox.ac.uk/crystal/refer.cgi?id=146641. The information about the death date and cause was collected from the certificates issued by the National Health Information Service (England and Wales) and the National Health Service Register for Scotland (Scotland). The outcomes were determined using the International Standard 《Classification of Diseases》, 10th edition (ICD-10) codes. The UK Biobank dataset provided an overview of all diagnoses and diagnosis dates, including the primary and secondary cause of death, ICD-10 diagnosis, and primary and secondary ICD-10 diagnosis. The main focus of the study was the outcomes of CVD mortality (ICD-I00-I99) and other CVD events, which included ASCVD (I70), CAD (I21–I23, I24.1, I25.2), PAD (I70, I74, I73.8, I73.9), and Heart Failure (I50). The study separately assessed ASCVD, CAD, PAD, and Heart Failure outcomes. (Detailed information is available at https://biobank.ctsu.ox.ac.uk/crystal/ukb/docs/DeathLinkage.pdf).

### Definition of covariates

Covariates were selected based on previous relevant studies. They were obtained at baseline by questionnaire or verbal interview, including age, gender, race, education, lifestyle habits [physical activity (IPAQ), smoke, drink], income, Townsend Deprivation Index (TDI), family history (hypertension, diabetes, heart disease), dietary status (dietary energy, dietary fiber, dietary quality), sleep status (chronotype, sleep duration, insomnia, snoring, daytime sleepiness) as well as the presence of sleep disorders, work system (shift work or not), self-reported illnesses and age at diagnosis of type 2 diabetes. In addition, we assessed participants’ diabetes severity status, metabolic control, and mental status (whether depressed or not). Definitions of higher severity of diabetes included diabetes duration ≥ 5 years, HbA1c ≥ 53 mmol/mol, diabetic complications, and insulin use. Sleep disorder outcomes were determined according to International Standard 《Classification of Diseases》 10th edition (ICD-10) codes (ICD-10/OPCS4-G47.0, F51.0, G47.1, G47.4, F51.1, G47.3, G47.2, F51.2. F51.3, F51.4, F51.5, G47.8, G47.9, F51.8, F51.9). Metabolic control was defined as the sum of metabolic risk factors exceeding the target range, including HbA1c (individualized metric), BP (total range < 140/90 mmHg), and LDL cholesterol (total range < 100 mg/dL). The CVD and T2DM standard PRS scores were created using a Bayesian method applied to meta-analyzed summary statistics GWAS data obtained from external GWAS data sources (the Standard PRS set). The Standard PRS set was calculated for all individuals in the UK Biobank. For additional variable definitions, please refer to the Additional file 1: Table S2.

### Statistical analyses

Baseline characteristics of 6860 participants with type 2 diabetes were statistically described according to three sleep patterns, with continuous variables using means ± standard deviation and number of cases and percentages for categorical variables. General linear models (for continuous variables) and chi-square tests (for categorical variables) were used to compare baseline characteristics among the three different sleep patterns. To assess the relationship between sleep patterns and (CVD) Mortality or other CVD events, multivariate COX Proportional Hazard Models (CPH) were employed. Hazard ratios (HRs) and 95% Confidence Intervals (CI) were used to determine the associations between each sleep pattern and CVD events. The analysis was conducted in four models. Model I was adjusted for age, gender, and race. Model II was further adjusted for BMI, smoking, drinking, physical activity, education, Thomson deprivation index (TDI), family history of diabetes, family history of hypertension, and family history of heart disease. Model III was additionally adjusted for dietary energy, dietary fiber, and dietary quality. Finally, Model IV was adjusted for age at diagnosis of type 2 diabetes mellitus and diabetes severity status. Sleep patterns were tested as a continuous variable to identify trends. Restricted cubic spline was used to test the linear relationship between sleep scores and CVD mortality, AS, CAD, PAD, and Heart Failure. The five sleep factors were stratified according to PRS tertiles, and multivariate COX regression models were developed. These models were then used to analyze the association between sleep patterns and the risk of CVD mortality, AS, CAD, PAD, and Heart Failure in the high, intermediate, and low genetic risk groups for each sleep factor. To test the robustness of our findings, we additionally performed the following sensitivity analyses: (1) Exclude individuals with sleep disorders, (2) excluded individuals with depression, (3) excluded individuals with shift work, (4) excluded individuals with severe state of type 2 diabetes mellitus, (5) stratified by age (≤ 60/> 60), gender (male/female), education (< high school/> high school), Thomson deprivation index (tertile), Age at diagnosis of T2DM (tertile), T2DM severity, metabolic control, CVD standard PRS (tertile) and T2DM standard PRS (tertile) and subgroups analyzed.

All statistical analyses were conducted using SPSS 25.0 and R software, and statistical significance was defined as a p-value < 0.05, two-tailed.

## Results

### Baseline characteristics

In Table 1, the baseline characteristics of diabetic patients are presented based on their sleep patterns. The study included 6860 participants, with 31.0% having poor sleep patterns, 39.1% having Intermediate sleep patterns, and 29.9% having healthy sleep patterns. Compared to those with poor sleep patterns, participants with healthy sleep patterns tended to be male, non-current smokers, highly physically active, and had lower Townsend Deprivation Index. Furthermore, participants with healthy sleep patterns had less Severity of type 2 diabetes and were less likely to suffer from depression.

**Table 1:** Baseline characteristics of participants with type 2 diabetes by sleep patterns

	Poor sleep	Moderate sleep	Sleep health	*P*
	N = 2127	N = 2685	N = 2048	
Survival time, per-month	148.51 (17.51)	149.21 (16.46)	149.4 (15.38)	0.080
Age, year	58.00 (7.55)	58.54 (7.39)	58.40 (7.40)	0.040
Male, n (%)	1182 (28.10)	1615 (38.40)	1410 (33.50)	0.000
White, n (%)	1922 (30.50)	2490 (39.50)	1896 (30.10)	0.011
BMI, kg/m^2^	30.19 (6.13)	29.89 (5.73)	30.11 (5.54)	0.120
College or higher, n (%)	1377 (30.40)	1773 (39.10)	1386 (30.60)	0.228
Current smoker, n (%)	211 (34.80)	234 (38.60)	161 (26.60)	0.207
Current drinker, n (%)	1870 (30.40)	2407 (39.20)	1866 (30.40)	0.019
IPAQ, n (%)				
Low	619 (35.20)	649 (36.90)	492 (28.00)	0.000
Moderate	862 (30.30)	1105 (38.80)	880 (30.90)	
High	646 (28.70)	931 (41.30)	676 (30.00)	
TDI	− 0.96 (3.15)	− 1.34 (3.01)	− 1.548 (2.84)	0.000
Energy, Kcal	2032.99 (588.26)	2044.06 (564.85)	2082.80 (567.98)	0.002
Fiber, g/day	17.76 (7.00)	18.14 (6.73)	18.21 (6.56)	0.000
Diet quality, n (%)				
0	25 (44.60)	24 (42.90)	7 (12.50)	0.010
1	586 (31.30)	695 (37.10)	594 (31.70)	
2	1138 (31.30)	1449 (39.80)	1051 (28.90)	
3	378 (29.30)	517 (40.00)	396 (30.70)	
Age at diagnosis of type 2 diabetes, year	56.30 (8.13)	57.06 (7.73)	56.94 (7.71)	0.000
Severity of type 2 diabetes, n (%)				
0	1070 (29.50)	1436 (39.60)	1117 (30.80)	0.004
1	659 (30.80)	841 (39.40)	637 (29.80)	
2	222 (34.00)	247 (37.90)	183 (28.10)	
3	55 (43.70)	48 (38.10)	23 (18.30)	
Family history of diabetes, n (%)	878 (31.50)	1083 (38.90)	822 (29.50)	0.717
Family history of hypertension, n (%)	1168 (32.80)	1380 (38.80)	1011 (28.40)	0.001
Family history of heart disease, n (%)	1095 (33.00)	1294 (39.00)	929 (28.00)	0.000
Sleep disorders, n (%)	166 (35.90)	172 (37.20)	124 (26.80)	0.054
Depression, n (%)	307 (45.20)	236 (34.80)	136 (20.00)	0.000
Shift work, n (%)	520 (33.40)	566 (36.30)	472 (30.30)	0.020
Chronotype, n (%)				
‘Morning’ person	301 (16.50)	731 (40.10)	791 (43.40)	0.000
‘Morning’ > evening	343 (14.40)	996 (41.90)	1039 (43.70)	
Evening < morning	1036 (54.60)	712 (37.50)	149 (7.90)	
‘Evening’ person	447 (58.70)	246 (32.30)	69 (9.10)	
Sleep duration, hours	6.75 (1.44)	7.30 (1.02)	7.58 (0.73)	0.000
Insomnia, n (%)				
Never/rarely	90 (5.20)	481 (27.80)	1162 (67.10)	0.000
Sometimes	1053 (34.80)	1388 (45.80)	589 (19.40)	
Usually	984 (46.90)	816 (38.90)	297 (14.20)	
Excessive daytime sleepiness, n (%)				
Never/rarely	1355 (28.40)	1919 (40.30)	1489 (31.30)	0.000
Sometimes	561 (31.00)	701 (38.80)	546 (30.20)	
Often	211 (73.00)	65 (22.50)	13 (4.50)	
Snoring, n (%)				
Yes	368 (12.00)	1112 (36.20)	1594 (51.90)	0.000
No	1759 (46.50)	1573 (41.50)	454 (12.00)	

The continuous variables were described with the mean (SD) and compared by a general linear model. The categorical variables were defined with n (%) and analyzed by the χ^2^. BMI, body mass index, kg/m^2^; IPAQ, International Physical Activity Questionnaire; TDI, Townsend deprivation index; SD: standard deviation.

### COX proportional risk model

**Fig. 2:**
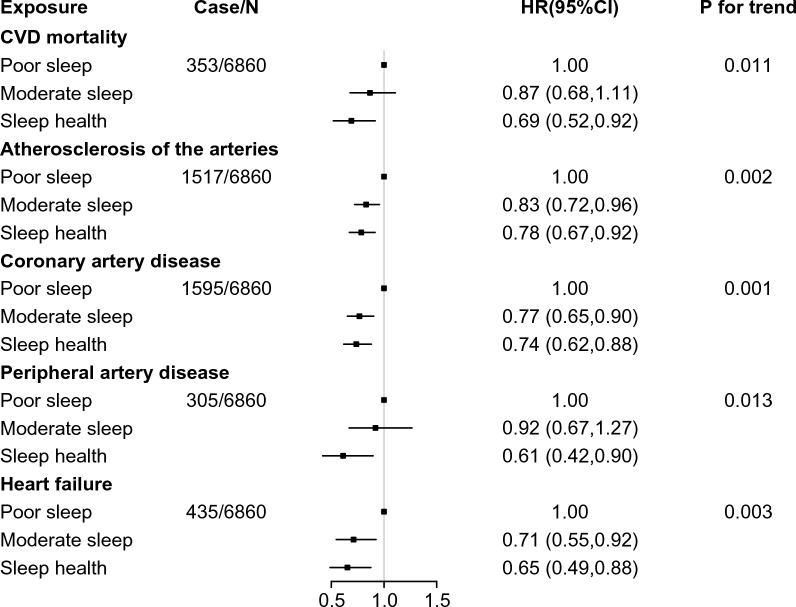
The risk of cardiovascular mortality and diseases among different sleep patterns participants. The multivariable model was adjusted for age, sex, race, BMI, smoking, drinking, sport, education, Thomson deprivation index, Family history of diabetes, Family history of hypertension, Family history of heart disease, and family history of heart disease, Dietary energy, Dietary fiber, Quality of diet, age at diagnosis of type 2 diabetes and Severity of diabetes. *CVD* cardiovascular disease, *CI* confidence interval, *HR* hazard ratio

We recorded 353 CVD Mortality and 1517 cases of ASCVD, 1595 cases of CAD, 305 cases of PAD, and 435 cases of Heart Failure in 6860 participants with Type 2 Diabetes Mellitus (T2DM). In a fully adjusted model (Model 4), using COX proportional hazard models, individuals with healthy sleep patterns were associated with a reduced risk of CVD Mortality and other CVD events compared with patients with poor sleep patterns [CVD Mortality (HR, 0.69; 95% CI 0.52–0.92), ASCVD (HR, 0.78; 95% CI 0.67–0.92), CAD (HR, 0.74; 95% CI 0.62–0.88), PAD (HR, 0.61; 95% CI 0.42–0.90), Heart Failure (HR, 0.65; 95% CI 0.49–0.88)]. However, no statistically significant association was found between Intermediate sleep patterns and reduced risk of CVD Mortality and PAD (Fig. 2) (Additional file 1: Table S3).

### Restricted cubic spline

**Fig. 3:**
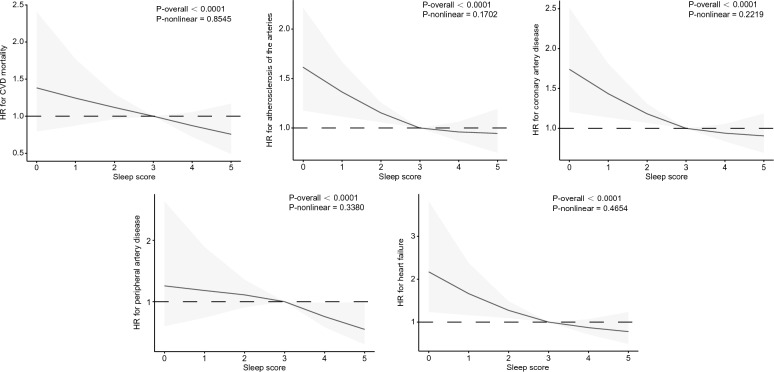
Associations between sleep score and CVD outcomes, including CVD mortality, ASCVD, CAD, PAD, and Heart Failure, were assessed using Cox proportional hazards regression models and restricted cubic splines. The models were adjusted for various factors such as age, sex, race, BMI, smoking, drinking, sport, education, Thomson deprivation index, family history of diabetes, hypertension, and heart disease, dietary energy, dietary fiber, quality of diet, age at diagnosis of type 2 diabetes, and severity of diabetes. Solid black lines represent Central estimates, while the gray-shaded areas represent the 95% confidence intervals

In Fig. 3, we examined the dose–response relationship between sleep score, CVD Mortality, and other CVD events using a Restricted Cubic Spline. The results showed a linear relationship between sleep score and CVD Mortality (P-nonlinear = 0.8545), ASCVD (P-nonlinear = 0.1702), CAD (P-nonlinear = 0.2219), PAD (P-nonlinear = 0.3380), Heart Failure (P-nonlinear = 0.4654) and had significant statistical differences with P-values < 0.0001. HR gradually decreased as sleep score increased. When Sleep Score = 3, HR = 1.

### Sleep PRS stratification analysis

We stratified by PRS tertiles of sleep factors and analyzed the association between sleep patterns and CVD in the low genetic risk, intermediate genetic risk, and high genetic risk groups for each sleep factor. Compared to individuals with poor sleep patterns, those with intermediate sleep patterns did not show any statistically significant difference in the association with reduced CVD Mortality and PAD in the fully adjusted model (p > 0.05). Surprisingly, in the high genetic risk group for snoring, intermediate sleep patterns were associated with a reduced risk of ASCVD and Heart Failure (HR: 0.625, 95% CI 0.483 to 0.807 and HR: 0.477, 95% CI 0.298 to 0.765, p < 0.05). Intermediate sleep patterns associated with reduced risk of CAD in high genetic risk groups for sleep duration, and snoring (HR: 0.611, 95% CI 0.410 to 0.912 and HR: 0.629, 95% CI 0.473 to 0.835, p < 0.05). What’s more, participants with healthy sleep patterns were significantly associated with reduced CVD Mortality in low genetic risk groups for chronotype, duration, insomnia, and Daytime Sleepiness (HR: 0.545, 95% CI 0.319 to 0.931, HR: 0.603, 95% CI 0.383 to 0.952, HR: 0.472, 95% CI 0.292 to 0.764, and HR: 0.538, 95% CI 0.308 to 0.941, p < 0.05 respectively). Additionally, individuals with high genetic risk for chronotype, insomnia, and snoring, who maintain healthy sleep patterns may have a lower risk of ASCVD (HR: 0.672, 95% CI 0.510 to 0.885, HR: 0.671, 95% CI 0.506 to 0.891, and HR: 0.666, 95% CI 0.506 to 0.876, p < 0.05 respectively); reduced risk of CAD in high genetic risk groups with healthy sleep patterns linked to chronotype, insomnia, daytime sleepiness, and snoring (HR: 0.701, 95% CI 0.515 to 0.954, HR: 0.623, 95% CI 0.454 to 0.854, HR: 0.689, 95% CI 0.503 to 0.943 and HR: 0.624, 95% CI 0.461 to 0.845, p < 0.05 respectively); healthy sleep patterns associated with reduced risk of PAD in high genetic risk group for chronotype, insomnia (HR: 0.443, 95% CI 0.231 to 0.852 and HR: 0.388, 95% CI 0.197 to 0.765, p < 0.05); healthy sleep patterns associated with reduced risk of Heart Failure in high genetic risk group for snoring (HR: 0.564, 95% CI 0.345 to 0.921, p < 0.05). (Additional file 1: Tables S4–S8).

### Sensitivity analysis

To further validate the association between sleep patterns and CVD Mortality, AS, CAD, PAD, and Heart Failure. We deleted people with sleep disorders, people with depression, people with shift work, and people with high diabetes severity. (Additional file 1: Tables S9–S12) The study used a multivariate COX proportional risk model and found that healthy sleep patterns are significantly associated with a lower risk of CVD Mortality, ASCVD, CAD, PAD, and Heart Failure. These results are consistent with earlier studies. Additionally, the trend test has a p-value < 0.05.

Finally, we conducted stratified analyses based on various factors such as age, sex, education, Thomson deprivation index, age at the diagnosis of type 2 diabetes, type 2 diabetes severity, metabolic control, CVD standard PRS, and T2DM standard PRS. We observed that the results were influenced by metabolic control and type 2 diabetes severity. Notably, metabolic control appeared to reduce the association between healthy sleep patterns and a lower risk of CVD mortality, ASCVD, CAD, PAD, and Heart Failure. Although there were no significant differences in CVD mortality, ASCVD, and CAD among different levels of type 2 diabetes severity, it was found that moderate and healthy sleep patterns were linked to Heart Failure in the group with higher severity of type 2 diabetes. (HR: 0.537, 95% CI 0.322 to 0.894, HR: 0.488, 95% CI 0.271 to 0.879, p < 0.05). CVD standard PRS and T2DM standard PRS were not significantly associated with reduced risk of CVD mortality, ASCVD, CAD, PAD, and Heart Failure. Otherwise, no substantial changes occurred in the other outcomes, especially in the strong association of healthy sleep patterns with reduced risk of CVD mortality, ASCVD, CAD, PAD, and Heart Failure among those aged > 60 years, male, with higher education, higher Townsend Deprivation Index and Age at diagnosis of type 2 diabetes > 54 years. (Additional file 1: Tables S13–S17).

## Discussion

In this large prospective cohort study, we analyzed the association of sleep patterns with CVD Mortality, ASCVD, CAD, PAD, and Heart Failure. A sleep score of 0–5 was obtained using five sleep factors (chronotype, sleep duration, insomnia, daytime sleepiness, and snoring), then three sleep patterns were constructed: a poor sleep pattern (0–2 points), an Intermediate sleep pattern (3–4 points) and a healthy sleep pattern (5 points). Compared to the poor sleep pattern, participants in the healthy sleep pattern had a 31%, 22%, 27%, 39%, and 35% lower risk of developing CVD Mortality, ASCVD, CAD, PAD, and Heart Failure, respectively. Under the conditions of the causal hypothesis, more than 20% of CVD Mortality and other CVD risk events would have been avoided if all five sleep factors had improved for all participants.

A systematic review of sleep disorders and the development of diabetes suggests that sleep disorders such as short (< 6 h) or long (> 9 h) sleep duration, insomnia, and obstructive sleep apnea (OSA) are consistent with traditional risk factors for diabetes. Therefore, sleep factors should be included in clinical guidelines for screening for type 2 diabetes [44]. A growing number of studies have demonstrated a higher risk of cardiovascular events and cardiovascular Mortality in patients with type 2 diabetes mellitus alone compared with nondiabetic patients [45]. In recent years, CVD has become the leading cause of death in patients with diabetes [46]. The link between sleep and CVD has been demonstrated in numerous studies. For instance, a cohort study utilizing the UKB database found that type 2 diabetic patients with less than 5 h of sleep or more than 10 h had an increased risk of ASCVD and CVD Mortality [47]. Another cohort study spanning 21 countries discovered that daytime sleepiness among people who slept more than 6 h at night increased the risk of CVD [33]. Additionally, a causal study based on Mendelian randomization found that sleep duration and insomnia were potential risk factors for CVD [48]. Lastly, a cohort study from a Chinese population over 40 found that poor sleep quality, snoring, and night shifts were associated with stroke risk factors [49]. Despite this evidence that good sleep is associated with a reduced risk of CVD, there are fewer studies on the relationship between sleep behaviors and CVD in people with type 2 diabetes. Related mechanistic studies have found that lipid distribution, chronic inflammation, glucose metabolism, and stress can partially explain the association between sleep factors and CVD. For example, compared with non-snoring populations, snoring populations have higher levels of body mass index (BMI), waist circumference, neck circumference, fasting blood glucose, triglycerides (TG), total cholesterol (TC), low-density lipoprotein (LDL), homocysteine (Hcy) and lower levels of high-density lipoprotein (HDL) (p < 0.05) [49]. In addition, the hypothalamus and the circadian rhythm control center, the sight cross nucleus (SCN), is the central clock of the circadian clock mechanism. The central clock and the peripheral clock located in the whole body jointly participate in the regulation of the sleep–wake cycle [50]. When sleep disorder is caused by sleep–wake cycle disorder, it will make people nervous and excited, the excitability of γ-aminobutyric acid (GABA) neurons is decreased, and the excitability of the cerebral cortex is increased, so that all levels of the central nervous system send vasoconstrictive impulses and sympathetic nerve activity is increased. Blood vessels are continuously in a state of contraction. Elevated blood pressure leads to high blood pressure, and if the blood flow continues to be poor, it may lead to endothelial damage, lipid accumulation, atherosclerosis, and coronary heart disease. Increased vascular pressure also leads to increased cardiac afterload, secondary arrhythmias, and compensatory myocardial hypertrophy. Increased vascular pressure also leads to increased cardiac afterload, secondary arrhythmias, and compensatory myocardial hypertrophy. The causes of sleep disorders leading to cardiovascular diseases include autonomic nervous balance disorder [51], changes in the circadian rhythm of peripheral vascular clock components, enhanced oxidative stress and induced inflammatory response, leading to reduced vascular endothelial function, increased vascular tone and decreased glucose tolerance, decreased insulin sensitivity, and increased body fat. However, the exact mechanism is currently unknown [52].

Research in the past has mainly focused on individual sleep factors. However, these factors are related to each other. For example, a study found a strong correlation between daytime sleepiness and insomnia. Individuals with insomnia have three times the risk of daytime sleepiness than those without insomnia [53]. Additionally, a sleep duration of less than six hours dramatically increases the risk of coronary heart disease in people with sleep disorders [54]. In this study, we used the sleep score created by Fan et al. to categorize the participants into three sleep patterns: poor sleep pattern (0–2 points), Intermediate sleep pattern (3–4 points), and healthy sleep pattern (5 points) [34]. Our findings are consistent with previous studies that evaluated the association between sleep patterns and CVD [34, 37, 55]. A study conducted in China revealed that maintaining healthy sleep patterns can significantly decrease the risk of cardiovascular diseases, coronary heart disease, and stroke [55]. Similarly, a study based on the UK Biobank database indicated that poor sleep patterns can worsen the risk of cardiovascular diseases, especially in individuals with poor glucose tolerance [56]. Therefore, our findings highlight the crucial role of healthy sleep patterns in reducing the incidence of CVD and related events among diabetic populations.

Based on the Restricted Cubic Spline (RCS) results, a linear relationship was found between sleep score and the risk of CVD mortality and other CVD events. As sleep scores increased, the risk of CVD Mortality and other specific CVD events gradually decreased. When sleep score = 3, HR = 1. This result is consistent with our definition of the three sleep patterns. Healthy Sleep Patterns (defined as early-rising chronotype; 7–9 h/day sleep duration; never or rarely insomnia; no snoring and low daytime sleepiness frequency) provide a positive sleep frame of reference for patients and is informative in identifying at-risk individuals and promoting health management. In addition, the RCS curve reveals that having at least three healthy sleep factors can significantly reduce the risk of CVD mortality, ASCVD, CAD, PAD, and Heart Failure. However, there was no significant difference between Intermediate sleep patterns and the risk of CVD Mortality and PAD. Nevertheless, healthy sleep patterns can still partially reduce the risk of CVD Mortality and PAD. These results remained consistent even after excluding those with sleep disorders, depression, night shifts, and severe diabetes. Hence, diabetic patients must manage their sleep more strictly to reduce the risk of CVD Mortality and PAD.

To further validate the outcomes, we conjectured whether the genetics of different sleep factors influenced sleep patterns. Then, we used sleep SNPs to calculate genetic risk scores for each sleep factor to observe whether the three groups of low genetic risk, intermediate genetic risk, and high genetic risk for sleep influence the relationship between sleep patterns and CVD risk. Our analysis showed no statistically significant difference in the association of Intermediate sleep patterns with CVD mortality, ASCVD, CAD, PAD, and Heart Failure among the three genetic risk groups for each sleep factor. Healthy sleep patterns are associated with reduced CVD mortality risk. However, this association was statistically significant only in the low genetic risk group for sleep factors. Furthermore, in the high genetic risk group for chronotype, insomnia, and snoring healthy sleep patterns were associated with a reduced risk of ASCVD and PAD. Healthy sleep patterns were also associated with a reduced risk of CAD in the high genetic risk group for daytime sleepiness, insomnia, and snoring and a reduced risk of Heart Failure in the high genetic risk group for chronotype and snoring [57].

Subgroup analysis revealed that a healthy sleep pattern can prevent metabolic control's negative impact on CVD death, ASCVD, CAD, PAD, and HF in diabetic patients. Moreover, studies have shown that diabetic patients with metabolic syndrome have a higher risk of cardiovascular disease [58]. However, healthy sleep patterns are only significantly associated with a reduced risk of heart failure in individuals with severe type 2 diabetes. Patients with type 2 diabetes and sleep disorders are prone to cardiac hypoxia, which exacerbates heart failure severity [59]. This also will provide compelling evidence for therapeutic interventions in diabetic cardiomyopathy (diabetic heart failure).

Our findings align with previous studies’ results and further emphasize the ability of healthy sleep patterns to mitigate the genetic influence of sleep factors. We offer valuable insights into optimizing sleep patterns for individuals with type 2 diabetes to mitigate their susceptibility to CVD mortality and CVD events. The findings also offer valuable guidance for future investigations into the mechanisms underlying cardiovascular disease in diabetic populations.

## Advantages and limitations

Our study has several strengths, including a prospective design based on a large sample size from the UKB database. As far as we know, our study is the first to systematically and comprehensively assess the relationship between sleep patterns and the risk of CVD mortality and other CVD events in diabetic patients. We used a restricted cubic spline to plot the relationship between sleep scores and the risk of CVD Mortality and other CVD events, which allows for a more visual representation of this relationship. In addition to this, we tested the relationship between sleep patterns and CVD risk in different genetic risk groups for sleep factors. However, there are also some limitations to our study. First, sleep factors are self-reported data, so misclassification and recall bias are inevitable. Misclassification may bias results in the direction of nullification and attenuation, thereby underestimating the association between exposure and outcome. Second, we turned the five sleep factors into dichotomous variables to obtain a sleep score of 0–5, potentially leading to missing information. The sleep score did not include all sleep factors, such as sleep deprivation being associated with an increased risk of cardiovascular disease [10]. Thirdly, we assessed sleep scores using baseline sleep characteristics and did not consider changes in sleep factors. Therefore, future studies could repeat the measurement of sleep factors and investigate the association with CVD before and after changes in sleep factors. Fourth, our study was based on a UK biobank, most of the patients were from Europe, which may affect the generalizability to other populations but does not affect the validity of the in-house study. Finally, although we have included most of the confounders, the presence of other unknown or unmeasured confounders remains possible.

## Conclusion

To summarize, this is an observational study that shows the benefits of a healthy sleep pattern with less frequencies of CVD events. It might reinforce the relevance of a healthy sleep pattern in this context, but the study does not have the power to recommend it as an intervention that will reverse the CVD risk—it is an extrapolation. But this study reinforce the relevance of future intervention studies that will provide more answers regarding this kind of recommendation.

### Supplementary Information

**Additional file 1: Table S1.** Original Questions for Self-Reported Sleep Characteristics. **Table S2.** Original Questions and Definitions for Covariates. **Table S3.** Association of sleep patterns with CVD events among people with type 2 diabetes. **Table S4.** Association of sleep patterns with CVD Mortality stratified by the PRS of Chronotype, Sleep duration, Insomnia, Daytime Sleepiness and Snoring. **Table S5.** Association of sleep patterns with ASCVD stratified by the PRS of Chronotype, Sleep duration, Insomnia, Daytime Sleepiness and Snoring. **Table S6.** Association of sleep patterns with CAD stratified by the PRS of Chronotype, Sleep duration, Insomnia, Daytime Sleepiness and Snoring. **Table S7.** Association of sleep patterns with PAD stratified by the PRS of Chronotype, Sleep duration, Insomnia, Daytime Sleepiness and Snoring. **Table S8.** Association of sleep patterns with Heart Failure stratified by the PRS Of Chronotype, Sleep duration, Insomnia, Daytime Sleepiness and Snoring. **Table S9.** Associations between sleep patterns and CVD events after excluding participants with sleep disorders. **Table S10.** Associations between sleep patterns and CVD events after excluding participants with Depression. **Table S11.** Associations between sleep patterns and CVD events after excluding participants on Shift. **Table S12.** Associations between sleep patterns and CVD events after excluding participants with Severe Diabetes. **Table S13.** Stratified analysis of the association between sleep patterns and CVD Mortality. **Table S14.** Stratified analysis of the association between sleep patterns and Atherosclerotic Cardiovascular Disease (ASCVD). **Table S15.** Stratified analysis of the association between sleep patterns and Coronary Artery Disease (CAD). **Table S16.** Stratified analysis of the association between sleep patterns and Peripheral Artery Disease (PAD). **Table S17.** Stratified analysis of the association between sleep patterns and Heart Failure.

**Additional file 2: Table S18.** Genome-wide association signals for Chronotype in participants of European ancestry from UK Biobank (n = 452,633). **Table S19.** Genome-wide association signals for sleep Duration in participants of European ancestry from UK Biobank (n = 452,633). **Table S20.** Genome-wide association signals for Insomnia in participants of European ancestry from UK Biobank (n = 452,633). **Table S21.** Genome-wide association signals for Daytime Napping in participants of European ancestry from UK Biobank (n = 452,633). **Table S22.** Genome-wide association signals for Snoring in participants of European ancestry from UK Biobank (n = 452,633).

## Data Availability

The datasets analyzed during the current study are available in the UK Biobank repository, https://www.ukbiobank.ac.uk/.

## Abbreviations

CVD: Cardiovascular disease
ASCVD: Atherosclerotic cardiovascular disease
CAD: Coronary artery disease
PAD: Peripheral artery disease
HF: Heart failure
BMI: Body mass index
PRS: Polygenic risk score
SNP: Single nucleotide polymorphism
IPAQ: International Physical Activity Questionnaire
TDI: Townsend deprivation index
TG: Triglycerides
TC: Total cholesterol
LDL: Low-density lipoprotein
HCY: Homocysteine
HDL: High-density lipoprotein
