# Supplementary material for: Sleep patterns and risks of incident cardiovascular disease and mortality among people with type 2 diabetes: a prospective study of the UK Biobank
Source: Diabetol Metab Syndr. 2024 Jan 11;16:15. doi: 10.1186/s13098-024-01261-8 (PMC10782582; doi:10.1186/s13098-024-01261-8)
Supplement: Supplementary file 1 — Additional file 1: Table S1. Original Questions for Self-Reported Sleep Characteristics. Table S2. Original Questions and Definitions for Covariates. Table S3. Association of sleep patterns with CVD events among people with type 2 diabetes. Table S4. Association of sleep patterns with CVD Mortality stratified by the PRS of Chronotype, Sleep duration, Insomnia, Daytime Sleepiness and Snoring. Table S5. Association of sleep patterns with ASCVD stratified by the PRS of Chronotype, Sleep duration, Insomnia, Daytime Sleepiness and Snoring. Table S6. Association of sleep patterns with CAD stratified by the PRS of Chronotype, Sleep duration, Insomnia, Daytime Sleepiness and Snoring. Table S7. Association of sleep patterns with PAD stratified by the PRS of Chronotype, Sleep duration, Insomnia, Daytime Sleepiness and Snoring. Table S8. Association of sleep patterns with Heart Failure stratified by the PRS Of Chronotype, Sleep duration, Insomnia, Daytime Sleepiness and Snoring. Table S9. Associations between sleep patterns and CVD events after excluding participants with sleep disorders. Table S10. Associations between sleep patterns and CVD events after excluding participants with Depression. Table S11. Associations between sleep patterns and CVD events after excluding participants on Shift. Table S12. Associations between sleep patterns and CVD events after excluding participants with Severe Diabetes. Table S13. Stratified analysis of the association between sleep patterns and CVD Mortality. Table S14. Stratified analysis of the association between sleep patterns and Atherosclerotic Cardiovascular Disease (ASCVD). Table S15. Stratified analysis of the association between sleep patterns and Coronary Artery Disease (CAD). Table S16. Stratified analysis of the association between sleep patterns and Peripheral Artery Disease (PAD). Table S17. Stratified analysis of the association between sleep patterns and Heart Failure. [file 13098_2024_1261_MOESM1_ESM.docx]

**Table S**1. Original Questions for Self-Reported Sleep Characteristics

| Characteristic | UK Biobank Code | UK Biobank Questionnaire | Healthy Answer | Unhealthy Answer |
| --- | --- | --- | --- | --- |
| Insomnia Complaint | 1200 | Do you have trouble falling asleep at night or do you wake up in the middle of the night? | Never/rarely; Sometimes | Usually |
| Sleep duration | 1160 | About how many hours sleep do you get in every 24 hours? (please include naps) (in integer). | 7 to <9 h/d. | <7 or >=9 h/d. |
| Snoring | 1210 | Does your partner or a close relative or friend complain about your Snoring? | No | Yes |
| Chronotype | 1180 | Do you consider yourself to be? | Definitely a “morning” person;  More a “morning” than “evening” person. | More an “evening” than a “morning person;  Definitely an “evening” person. |
| Daytime Sleepiness | 1220 | How likely are you to doze off or fall asleep during the daytime when you don't mean to? (e.g. when working, reading or driving) | Never/rarely; Sometimes | Often; All the Time |

Table S**2.** Original Questions and Definitions for Covariates

| Variable | UK Biobank Code | UK Biobank Questionnaire/Definition | Modification in the Present Study | Category |
| --- | --- | --- | --- | --- |
| Socioeconomic status (Townsend deprivation index) | 189 | Each participant is assigned a score corresponding to the output area in which their postcode is located. The higher the score, the more deprived the area. | Derived into quantiles. | 1^st^ quantile;  2^nd^ quantile;  3^rd^ quantile;  4^th^ quantile. |
| Mental health  issue | 2090, 2100 | Have you ever seen a general practitioner (GP)/ psychiatrist for nerves, anxiety, tension or depression? | If either is true then true. | Yes;  No |
| Body mass index (BMI) | 21001 | BMI value here is constructed from height and weight measured during the initial Assessment Centre visit. | Derived into three groups. | <30;  30 to <40;  >= 40 |
| Cigarette smoking | 20116 | This field summarizes the current/past smoking status of the participant. | NA | Never;  Previous smoker; Current smoker |
| Alcohol consumption | 1558, 1568, 1578, 1588, 1598, 1608, 5364, 20117 | The level of overall alcohol consumption as the number of UK units of alcohol (10 mL/unit) consumed per week was calculated; participants were categorized based on the consumption according to the UK guideline (14 UK units/wk). See <https://biobank.ndph.ox.ac.uk/showcase/label.cgi?id=100051> for full questionnaire details. | | Never;  Previous drinker;  Within guidelines (14 units/wk) (including occasional drinkers);  Above guidelines (14-28 units/wk);  Double guidelines (>28 units/wk). |
| Diet quality | 1289, 1299,  1309, 1319,  1329, 1339,  1349, 1369,  1379, 1389 | Four healthy diet patterns were evaluated based on the American Heart Asociation (AHA), >4.5 servings/d fruits and vegetables, >= 2 times/wk fish intake, <2 times/wk processed meat, <5 times/wk red meat intake. Participants got one score for each criterion. We then categorized them based on the score. See <https://biobank.ndph.ox.ac.uk/showcase/label.cgi?id=100052> for full questionnaire details. | | Poor (<= 1 score);  Intermediate (2 to 3 score);  Healthy (> 4 score). |
| Physical activity | 864, 874, 884, 894, 904, 914 | The UK Biobank applied a the modified-version short-form International Physical Activity Questionnaire (IPAQ). We summarized weekly PA using weekly total Metabolic Equivalent Task (MET), calculated by multiplying the MET value of activity by the number of PA hours per week. Based on the lower and upper limits of the World Health Organization (WHO) PA guideline, we categorized PA into three groups. Another category “No moderate-to-vigorous PA” was further defined. See <https://biobank.ndph.ox.ac.uk/showcase/label.cgi?id=100054> for full questionnaire details. | | No moderate-to-vigorous PA;  Low (0 to < 600 MET-mins/wk); Medium (600 to < 1200 MET-mins/wk);  High (≥ 1200 MET-mins/wk). |

| **Table S3**. Association of sleep patterns with CVD events among people with type 2 diabetes | | | | | | | |
| --- | --- | --- | --- | --- | --- | --- | --- |
|  |  | Poor sleep | Intermediate sleep | | Sleep health | | P_trend_ |
|  |  |  | HR | 95% CI | HR | 95% CI |  |
| CVD mortality |  |  |  |  |  |  |  |
|  | Model 1^a^ | Ref (1) | **0.776** | **(0.610,0.985)** | **0.586** | **(0.445,0.771)** | **0.000** |
|  | Model 2 | Ref (1) | 0.842 | (0.662,1.071) | **0.655** | **(0.496,0.864)** | **0.003** |
|  | Model 3 | Ref (1) | 0.848 | (0.666,1.079) | **0.658** | **(0.498,0.869)** | **0.003** |
|  | Model 4 | Ref (1) | 0.867 | (0.678,1.108) | **0.69** | **(0.519,0.916)** | **0.011** |
| AS |  |  |  |  |  |  |  |
|  | Model 1 | Ref (1) | **0.74** | **(0.646,0.849)** | **0.678** | **(0.584,0.787)** | **0.000** |
|  | Model 2 | Ref (1) | **0.786** | **(0.685,0.902)** | **0.746** | **(0.642,0.867)** | **0.000** |
|  | Model 3 | Ref (1) | **0.788** | **(0.687,0.904)** | **0.749** | **(0.644,0.870)** | **0.000** |
|  | Model 4 | Ref (1) | **0.83** | **(0.721,0.956)** | **0.784** | **(0.671,0.915)** | **0.002** |
| CAD |  |  |  |  |  |  |  |
|  | Model 1 | Ref (1) | **0.705** | **(0.602,0.826)** | **0.678** | **(0.573,0.803)** | **0.000** |
|  | Model 2 | Ref (1) | **0.731** | **(0.624,0.856)** | **0.718** | **(0.606,0.851)** | **0.000** |
|  | Model 3 | Ref (1) | **0.734** | **(0.626,0.86)** | **0.724** | **(0.611,0.858)** | **0.000** |
|  | Model 4 | Ref (1) | **0.767** | **(0.653,0.902)** | **0.737** | **(0.618,0.879)** | **0.001** |
| PAD |  |  |  |  |  |  |  |
|  | Model 1 | Ref (1) | 0.79 | (0.579,1.078) | **0.547** | **(0.380,0.786)** | **0.001** |
|  | Model 2 | Ref (1) | 0.852 | (0.623,1.165) | **0.613** | **(0.425,0.884)** | **0.009** |
|  | Model 3 | Ref (1) | 0.867 | (0.634,1.186)) | **0.616** | **(0.427,0.888)** | **0.010** |
|  | Model 4 | Ref (1) | 0.919 | (0.669,1.265) | **0.612** | **(0.418,0.896)** | **0.013** |
| HF |  |  |  |  |  |  |  |
|  | Model 1 | Ref (1) | **0.629** | **(0.487,0.813)** | **0.578** | **(0.437,0.765)** | **0.000** |
|  | Model 2 | Ref (1) | **0.667** | **(0.516,0.862)** | **0.628** | **(0.474,0.834)** | **0.001** |
|  | Model 3 | Ref (1) | **0.669** | **(0.517,0.865)** | **0.631** | **(0.476,0.837)** | **0.001** |
|  | Model 4 | Ref (1) | **0.711** | **(0.547,0.924)** | **0.653** | **(0.488,0.875)** | **0.003** |

^a^Model 1 were adjusted for age, sex, race. Model 2 further adjusted for BMI, smoke, drink, sport, education, Thomson index, Family history of diabetes, Family history of hypertension, Family history of heart disease. Model 3 further adjusted for Dietary energy, Dietary fiber, Quality of diet. Model 4 further adjusted for Age at diagnosis of type 2 diabetes, Severity of diabetes.

CVD: cardiovascular disease; ASCVD: atherosclerosis cardiovascular disease; CAD: coronary artery disease PAD: peripheral arterial disease; HF: Heart Failure; Ref, reference; HR: hazard ratio; CI: confidence interval.

| **Table S4.** Association of sleep patterns with CVD Mortality stratified by the PRS of Chronotype, Sleep duration, Insomnia, Daytime Sleepiness and Snoring. | | | | | | | | |
| --- | --- | --- | --- | --- | --- | --- | --- | --- |
|  |  | Tertiles | Poor sleep | Intermediate sleep | | Sleep health | | P_trend_ |
|  |  |  |  | HR | 95% CI | HR | 95% CI |  |
| Chronotype | |  |  |  |  |  |  |  |
|  | Model 1^a^ | ≤ -0.00006 | Ref (1) | 0.889 | (0.597,1.325) | **0.480** | **(0.286,0.805)** | **0.007** |
|  |  | -0.00006 - 0.00118 | Ref (1) | 0.773 | (0.498,1.199) | 0.632 | (0.390,1.026) | 0.062 |
|  |  | ≥ 0.00118 | Ref (1) | 0.677 | (0.434,1.055) | **0.604** | **(0.371,0.982)** | **0.040** |
|  | Model 2 | ≤ -0.00006 | Ref (1) | 0.990 | (0.659,1.486) | **0.535** | **(0.317,0.903)** | **0.027** |
|  |  | -0.00006 - 0.00118 | Ref (1) | 0.838 | (0.538,1.306) | 0.750 | (0.459,1.227) | 0.248 |
|  |  | ≥ 0.00118 | Ref (1) | 0.705 | (0.451,1.104) | 0.637 | (0.390,1.041) | 0.070 |
|  | Model 3 | ≤ -0.00006 | Ref (1) | 1.009 | (0.670,1.518) | **0.538** | **(0.318,0.909)** | **0.030** |
|  |  | -0.00006 - 0.00118 | Ref (1) | 0.854 | (0.548,1.331) | 0.761 | (0.465,1.246) | 0.274 |
|  |  | ≥ 0.00118 | Ref (1) | 0.716 | (0.457,1.122) | 0.629 | (0.385,1.030) | 0.063 |
|  | Model 4 | ≤ -0.00006 | Ref (1) | 1.027 | (0.679,1.553) | **0.545** | **(0.319,0.931)** | **0.039** |
|  |  | -0.00006 - 0.00118 | Ref (1) | 0.893 | (0.569,1.402) | 0.757 | (0.454,1.260) | 0.284 |
|  |  | ≥ 0.00118 | Ref (1) | 0.720 | (0.457,1.135) | 0.674 | (0.410,1.109) | 0.116 |
| Sleep duration | |  |  |  |  |  |  |  |
|  | Model 1 | ≤ 0 .01256 | Ref (1) | 0.777 | (0.537,1.126) | **0.508** | **(0.325,0.794)** | **0.003** |
|  |  | 0.01256 - 0.01624 | Ref (1) | 0.745 | (0.502,1.106) | **0.601** | **(0.388,0.930)** | **0.021** |
|  |  | ≥ 0.01624 | Ref (1) | 0.817 | (0.448,1.488) | 0.673 | (0.337,1.344) | 0.259 |
|  | Model 2 | ≤ 0 .01256 | Ref (1) | 0.814 | (0.561,1.181) | **0.562** | **(0.358,0.882)** | **0.012** |
|  |  | 0.01256 - 0.01624 | Ref (1) | 0.797 | (0.536,1.187) | 0.658 | (0.423,1.022) | 0.061 |
|  |  | ≥ 0.01624 | Ref (1) | 0.930 | (0.500,1.731) | 0.779 | (0.384,1.578) | 0.490 |
|  | Model 3 | ≤ 0 .01256 | Ref (1) | 0.821 | (0.565,1.193) | **0.565** | **(0.360,0.888)** | **0.013** |
|  |  | 0.01256 - 0.01624 | Ref (1) | 0.793 | (0.532,1.183) | 0.656 | (0.421,1.020) | 0.060 |
|  |  | ≥ 0.01624 | Ref (1) | 0.981 | (0.525,1.835) | 0.817 | (0.402,1.660) | 0.581 |
|  | Model 4 | ≤ 0 .01256 | Ref (1) | 0.842 | (0.578,1.227) | **0.603** | **(0.383,0.952)** | **0.031** |
|  |  | 0.01256 - 0.01624 | Ref (1) | 0.792 | (0.527,1.189) | 0.637 | (0.403,1.006) | 0.051 |
|  |  | ≥ 0.01624 | Ref (1) | 1.071 | (0.574,2.000) | 0.891 | (0.436,1.824) | 0.769 |
| Insomnia | |  |  |  |  |  |  |  |
|  | Model 1 | ≤ -0.00526 | Ref (1) | 0.867 | (0.597,1.257) | **0.446** | **(0.279,0.712)** | **0.001** |
|  |  | -0.00526 - -0.00269 | Ref (1) | 0.719 | (0.463,1.115) | 0.758 | (0.469,1.224) | 0.231 |
|  |  | ≥ -0.00269 | Ref (1) | 0.727 | (0.443,1.193) | 0.594 | (0.346,1.020) | 0.057 |
|  | Model 2 | ≤ -0.00526 | Ref (1) | 0.889 | (0.610,1.295) | **0.468** | **(0.291,0.751)** | **0.002** |
|  |  | -0.00526 - -0.00269 | Ref (1) | 0.829 | (0.531,1.294) | 0.927 | (0.568,1.514) | 0.716 |
|  |  | ≥ -0.00269 | Ref (1) | 0.749 | (0.456,1.232) | 0.636 | (0.369,1.098) | 0.100 |
|  | Model 3 | ≤ -0.00526 | Ref (1) | 0.887 | (0.608,1.293) | **0.470** | **(0.292,0.755)** | **0.002** |
|  |  | -0.00526 - -0.00269 | Ref (1) | 0.843 | (0.539,1.320) | 0.938 | (0.574,1.534) | 0.757 |
|  |  | ≥ -0.00269 | Ref (1) | 0.748 | (0.455,1.230) | 0.633 | (0.366,1.093) | 0.097 |
|  | Model 4 | ≤ -0.00526 | Ref (1) | 0.909 | (0.621,1.332) | **0.472** | **(0.292,0.764)** | **0.003** |
|  |  | -0.00526 - -0.00269 | Ref (1) | 0.910 | (0.578,1.431) | 0.994 | (0.601,1.643) | 0.951 |
|  |  | ≥ -0.00269 | Ref (1) | 0.686 | (0.413,1.139) | 0.638 | (0.366,1.111) | 0.104 |
| Daytime Sleepiness | |  |  |  |  |  |  |  |
|  | Model 1 | ≤ 0.00587 | Ref (1) | 0.766 | (0.498,1.179) | **0.471** | **(0.278,0.799)** | **0.005** |
|  |  | 0.0058 - 0.00676 | Ref (1) | **0.642** | **(0.418,0.985)** | **0.505** | **(0.308,0.827)** | **0.005** |
|  |  | ≥ 0.00676 | Ref (1) | 0.954 | (0.626,1.456) | 0.766 | (0.480,1.224) | 0.265 |
|  | Model 2 | ≤ 0.00587 | Ref (1) | 0.838 | (0.543,1.294) | **0.551** | **(0.322,0.942)** | **0.031** |
|  |  | 0.0058 - 0.00676 | Ref (1) | 0.688 | (0.446,1.060) | **0.585** | **(0.355,0.963)** | **0.029** |
|  |  | ≥ 0.00676 | Ref (1) | 1.032 | (0.674,1.580) | 0.816 | (0.509,1.310) | 0.405 |
|  | Model 3 | ≤ 0.00587 | Ref (1) | 0.851 | (0.551,1.314) | **0.555** | **(0.324,0.949)** | **0.034** |
|  |  | 0.0058 - 0.00676 | Ref (1) | 0.697 | (0.450,1.078) | **0.589** | **(0.357,0.971)** | **0.032** |
|  |  | ≥ 0.00676 | Ref (1) | 1.020 | (0.666,1.563) | 0.811 | (0.504,1.304) | 0.392 |
|  | Model 4 | ≤ 0.00587 | Ref (1) | 0.872 | (0.563,1.351) | **0.538** | **(0.308,0.941)** | **0.034** |
|  |  | 0.0058 - 0.00676 | Ref (1) | 0.720 | (0.465,1.117) | **0.598** | **(0.362,0.987)** | **0.039** |
|  |  | ≥ 0.00676 | Ref (1) | 1.023 | (0.663,1.580) | 0.876 | (0.540,1.423) | 0.602 |
| Snoring | |  |  |  |  |  |  |  |
|  | Model 1 | ≤ 0.00020 | Ref (1) | 0.846 | (0.558,1.284) | 0.657 | (0.408,1.059) | 0.084 |
|  |  | 0.00020 - 0.00111 | Ref (1) | 0.849 | (0.566,1.272) | **0.545** | **(0.337,0.883)** | **0.014** |
|  |  | ≥ 0.00111 | Ref (1) | 0.630 | (0.395,1.005) | **0.526** | **(0.312,0.886)** | **0.013** |
|  | Model 2 | ≤ 0.00020 | Ref (1) | 0.966 | (0.633,1.475) | 0.743 | (0.459,1.202) | 0.236 |
|  |  | 0.00020 - 0.00111 | Ref (1) | 0.870 | (0.579,1.308) | **0.613** | **(0.377,0.997)** | 0.051 |
|  |  | ≥ 0.00111 | Ref (1) | 0.674 | (0.420,1.081) | **0.578** | **(0.340,0.984)** | **0.038** |
|  | Model 3 | ≤ 0.00020 | Ref (1) | 0.964 | (0.631,1.472) | 0.742 | (0.459,1.202) | 0.235 |
|  |  | 0.00020 - 0.00111 | Ref (1) | 0.889 | (0.589,1.342) | **0.613** | **(0.377,0.997)** | 0.051 |
|  |  | ≥ 0.00111 | Ref (1) | 0.667 | (0.415,1.072) | 0.591 | (0.347,1.008) | **0.046** |
|  | Model 4 | ≤ 0.00020 | Ref (1) | 0.969 | (0.634,1.483) | 0.751 | (0.461,1.222) | 0.261 |
|  |  | 0.00020 - 0.00111 | Ref (1) | 0.911 | (0.597,1.388) | 0.632 | (0.384,1.040) | 0.075 |
|  |  | ≥ 0.00111 | Ref (1) | 0.704 | (0.435,1.140) | 0.617 | (0.358,1.064) | 0.073 |

^a^Model 1 were adjusted for age, sex, race. Model 2 further adjusted for BMI, smoke, drink, sport, education, Thomson index, Family history of diabetes, Family history of hypertension, Family history of heart disease. Model 3 further adjusted for Dietary energy, Dietary fiber, Quality of diet. Model 4 further adjusted for Age at diagnosis of type 2 diabetes, Severity of diabetes.

Ref, reference; HR: hazard ratio; CI: confidence interval.

| **Table S5.** Association of sleep patterns with ASCVD stratified by the PRS of Chronotype, Sleep duration, Insomnia, Daytime Sleepiness and Snoring. | | | | | | | | |
| --- | --- | --- | --- | --- | --- | --- | --- | --- |
|  |  | Tertiles | Poor sleep | Intermediate sleep | | Sleep health | | P_trend_ |
|  |  |  |  | HR | 95% CI | HR | 95% CI |  |
| Chronotype | | |  |  |  |  |  |  |
|  | Model 1^a^ | ≤ -0.00006 | Ref (1) | **0.696** | **(0.548,0.883)** | **0.654** | **(0.501,0.855)** | **0.001** |
|  |  | -0.00006 - 0.00119 | Ref (1) | **0.713** | **(0.552,0.920)** | **0.724** | **(0.556,0.942)** | **0.017** |
|  |  | 0.00119 | Ref (1) | **0.725** | **(0.570,0.922)** | **0.605** | **(0.464,0.789)** | **0.000** |
|  | Model 2 | ≤ -0.00006 | Ref (1) | **0.728** | **(0.573,0.926)** | **0.704** | **(0.537,0.922)** | **0.007** |
|  |  | -0.00006 - 0.00119 | Ref (1) | 0.812 | (0.627,1.051) | 0.858 | (0.656,1.123) | 0.264 |
|  |  | 0.00119 | Ref (1) | **0.754** | **(0.592,0.960)** | **0.637** | **(0.487,0.833)** | **0.001** |
|  | Model 3 | ≤ -0.00006 | Ref (1) | **0.747** | **(0.587,0.951)** | **0.716** | **(0.546,0.938)** | **0.011** |
|  |  | -0.00006 - 0.00119 | Ref (1) | 0.816 | (0.630,1.057) | 0.852 | (0.651,1.115) | 0.244 |
|  |  | 0.00119 | Ref (1) | **0.751** | **(0.590,0.957)** | **0.635** | **(0.485,0.831)** | **0.001** |
|  | Model 4 | ≤ -0.00006 | Ref (1) | 0.803 | (0.628,1.026) | **0.734** | **(0.555,0.969)** | **0.024** |
|  |  | -0.00006 - 0.00119 | Ref (1) | 0.82 | (0.631,1.066) | 0.872 | (0.663,1.147) | 0.325 |
|  |  | 0.00119 | Ref (1) | 0.814 | (0.635,1.042) | **0.672** | **(0.510,0.885)** | **0.005** |
| Sleep duration | | |  |  |  |  |  |  |
|  | Model 1 | ≤ 0.01256 | Ref (1) | **0.747** | **(0.602,0.926)** | **0.570** | **(0.446,0.729)** | **0.000** |
|  |  | 0.01256 - 0.01624 | Ref (1) | **0.746** | **(0.596,0.934)** | **0.678** | **(0.534,0.862)** | **0.002** |
|  |  | ≥ 0.01624 | Ref (1) | **0.590** | **(0.422,0.826)** | 0.822 | (0.586,1.154) | 0.243 |
|  | Model 2 | ≤ 0.01256 | Ref (1) | **0.774** | **(0.624,0.961)** | **0.634** | **(0.494,0.813)** | **0.000** |
|  |  | 0.01256 - 0.01624 | Ref (1) | 0.820 | (0.654,1.028) | **0.745** | **(0.585,0.949)** | **0.017** |
|  |  | ≥ 0.01624 | Ref (1) | **0.639** | **(0.453,0.900)** | 0.923 | (0.655,1.302) | 0.611 |
|  | Model 3 | ≤ 0.01256 | Ref (1) | **0.774** | **(0.624,0.961)** | **0.630** | **(0.491,0.808)** | **0.000** |
|  |  | 0.01256 - 0.01624 | Ref (1) | 0.815 | (0.649,1.022) | **0.742** | **(0.582,0.946)** | **0.016** |
|  |  | ≥ 0.01624 | Ref (1) | **0.640** | **(0.453,0.902)** | 0.938 | (0.664,1.326) | 0.679 |
|  | Model 4 | ≤ 0.01256 | Ref (1) | **0.787** | **(0.632,0.978)** | **0.629** | **(0.487,0.812)** | **0.000** |
|  |  | 0.01256 - 0.01624 | Ref (1) | 0.841 | (0.667,1.060) | **0.764** | **(0.596,0.980)** | **0.034** |
|  |  | ≥ 0.01624 | Ref (1) | 0.767 | (0.538,1.093) | 1.075 | (0.751,1.537) | 0.711 |
| Insomnia | |  |  |  |  |  |  |  |
|  | Model 1 | ≤ -0.00522 | Ref (1) | 0.833 | (0.654,1.063) | **0.698** | **(0.536,0.910)** | **0.008** |
|  |  | -0.00522 - -0.00264 | Ref (1) | **0.572** | **(0.450,0.728)** | **0.670** | **(0.519,0.866)** | **0.001** |
|  |  | ≥ -0.00264 | Ref (1) | **0.777** | **(0.604,0.998)** | **0.625** | **(0.475,0.822)** | **0.001** |
|  | Model 2 | ≤ -0.00522 | Ref (1) | 0.885 | (0.693,1.131) | **0.759** | **(0.580,0.993)** | **0.044** |
|  |  | -0.00522 - -0.00264 | Ref (1) | **0.609** | **(0.477,0.777)** | **0.750** | **(0.578,0.974)** | **0.016** |
|  |  | ≥ -0.00264 | Ref (1) | 0.797 | (0.620,1.025) | **0.664** | **(0.503,0.876)** | **0.004** |
|  | Model 3 | ≤ -0.00522 | Ref (1) | 0.883 | (0.690,1.129) | **0.762** | **(0.582,0.998)** | **0.048** |
|  |  | -0.00522 - -0.00264 | Ref (1) | **0.616** | **(0.482,0.787)** | **0.755** | **(0.581,0.981)** | **0.020** |
|  |  | ≥ -0.00264 | Ref (1) | 0.795 | (0.618,1.023) | **0.665** | **(0.504,0.877)** | **0.004** |
|  | Model 4 | ≤ -0.00522 | Ref (1) | 0.932 | (0.725,1.198) | 0.785 | (0.596,1.035) | 0.087 |
|  |  | -0.00522 - -0.00264 | Ref (1) | **0.666** | **(0.518,0.856)** | 0.810 | (0.620,1.058) | 0.086 |
|  |  | ≥ -0.00264 | Ref (1) | 0.807 | (0.625,1.042) | **0.671** | **(0.506,0.891)** | **0.006** |
| Daytime Sleepiness | | |  |  |  |  |  |  |
|  | Model 1 | ≤ 0.00587 | Ref (1) | **0.708** | **(0.555,0.904)** | **0.605** | **(0.461,0.795)** | **0.000** |
|  |  | 0.00587 - 0.00675 | Ref (1) | **0.612** | **(0.477,0.784)** | **0.660** | **(0.509,0.856)** | **0.002** |
|  |  | ≥ 0.00675 | Ref (1) | 0.845 | (0.664,1.074) | **0.722** | **(0.556,0.938)** | **0.014** |
|  | Model 2 | ≤ 0.00587 | Ref (1) | **0.755** | **(0.591,0.965)** | **0.695** | **(0.527,0.917)** | **0.008** |
|  |  | 0.00587 - 0.00675 | Ref (1) | **0.651** | **(0.507,0.836)** | **0.733** | **(0.563,0.955)** | **0.019** |
|  |  | ≥ 0.00675 | Ref (1) | 0.897 | (0.703,1.143) | 0.773 | (0.594,1.007) | 0.056 |
|  | Model 3 | ≤ 0.00587 | Ref (1) | **0.767** | **(0.600,0.980)** | **0.698** | **(0.529,0.922)** | **0.009** |
|  |  | 0.00587 - 0.00675 | Ref (1) | **0.664** | **(0.516,0.854)** | **0.739** | **(0.567,0.962)** | **0.022** |
|  |  | ≥ 0.00675 | Ref (1) | 0.891 | (0.699,1.137) | 0.772 | (0.592,1.006) | 0.056 |
|  | Model 4 | ≤ 0.00587 | Ref (1) | **0.751** | **(0.586,0.963)** | **0.697** | **(0.524,0.926)** | **0.010** |
|  |  | 0.00587 - 0.00675 | Ref (1) | **0.710** | **(0.550,0.917)** | **0.755** | **(0.576,0.989)** | **0.038** |
|  |  | ≥ 0.00675 | Ref (1) | 1.000 | (0.778,1.285) | 0.844 | (0.642,1.109) | 0.229 |
| Snoring | |  |  |  |  |  |  |  |
|  | Model 1 | ≤ 0.00020 | Ref (1) | 0.911 | (0.725,1.145) | 0.801 | (0.623,1.030) | 0.084 |
|  |  | 0.00020 - 0.00111 | Ref (1) | **0.703** | **(0.542,0.913)** | **0.606** | **(0.457,0.805)** | **0.000** |
|  |  | ≥ 0.00111 | Ref (1) | **0.551** | **(0.429,0.707)** | **0.590** | **(0.454,0.768)** | **0.000** |
|  | Model 2 | ≤ 0.00020 | Ref (1) | 0.983 | (0.781,1.238) | 0.901 | (0.698,1.162) | 0.428 |
|  |  | 0.00020 - 0.00111 | Ref (1) | **0.728** | **(0.560,0.946)** | **0.648** | **(0.487,0.861)** | **0.003** |
|  |  | ≥ 0.00111 | Ref (1) | **0.594** | **(0.461,0.766)** | **0.664** | **(0.508,0.868)** | **0.002** |
|  | Model 3 | ≤ 0.00020 | Ref (1) | 0.987 | (0.784,1.242) | 0.907 | (0.703,1.170) | 0.460 |
|  |  | 0.00020 - 0.00111 | Ref (1) | **0.739** | **(0.567,0.961)** | **0.653** | **(0.491,0.869)** | **0.003** |
|  |  | ≥ 0.00111 | Ref (1) | **0.594** | **(0.461,0.766)** | **0.669** | **(0.512,0.874)** | **0.002** |
|  | Model 4 | ≤ 0.00020 | Ref (1) | 1.041 | (0.824,1.317) | 0.962 | (0.741,1.249) | 0.790 |
|  |  | 0.00020 - 0.00111 | Ref (1) | 0.764 | (0.583,1.002) | **0.685** | **(0.512,0.918)** | **0.011** |
|  |  | ≥ 0.00111 | Ref (1) | **0.625** | **(0.483,0.807)** | **0.666** | **(0.506,0.876)** | **0.002** |

^a^Model 1 were adjusted for age, sex, race. Model 2 further adjusted for BMI, smoke, drink, sport, education, Thomson index, Family history of diabetes, Family history of hypertension, Family history of heart disease. Model 3 further adjusted for Dietary energy, Dietary fiber, Quality of diet. Model 4 further adjusted for Age at diagnosis of type 2 diabetes, Severity of diabetes.

Ref, reference; HR: hazard ratio; CI: confidence interval.

| **Table S6.** Association of sleep patterns with CAD stratified by the PRS of Chronotype, Sleep duration, Insomnia, Daytime Sleepiness and Snoring. | | | | | | | | |
| --- | --- | --- | --- | --- | --- | --- | --- | --- |
|  |  | Tertiles | Poor sleep | Intermediate sleep | | Sleep health | | P_trend_ |
|  |  |  |  | HR | 95% CI | HR | 95% CI |  |
| Chronotype | | | | | | | | |
|  | Model 1^a^ | ≤ -0.00005 | Ref (1) | **0.540** | **(0.407,0.719)** | **0.592** | **(0.436,0.805)** | **0.000** |
|  |  | -0.00005 - 0.00119 | Ref (1) | 0.844 | (0.638,1.116) | **0.698** | **(0.516,0.944)** | **0.019** |
|  |  | ≥ 0.00119 | Ref (1) | **0.724** | **(0.547,0.957)** | **0.686** | **(0.510,0.924)** | **0.014** |
|  | Model 2 | ≤ -0.00005 | Ref (1) | **0.560** | **(0.421,0.746)** | **0.620** | **(0.455,0.845)** | **0.001** |
|  |  | -0.00005 - 0.00119 | Ref (1) | 0.891 | (0.672,1.182) | 0.758 | (0.559,1.029) | 0.075 |
|  |  | ≥ 0.00119 | Ref (1) | **0.732** | **(0.552,0.970)** | **0.703** | **(0.521,0.949)** | **0.023** |
|  | Model 3 | ≤ -0.00005 | Ref (1) | **0.579** | **(0.434,0.773)** | **0.636** | **(0.466,0.868)** | **0.002** |
|  |  | -0.00005 - 0.00119 | Ref (1) | 0.902 | (0.680,1.197) | 0.755 | (0.556,1.025) | 0.072 |
|  |  | ≥ 0.00119 | Ref (1) | **0.724** | **(0.546,0.961)** | **0.703** | **(0.521,0.949)** | **0.023** |
|  | Model 4 | ≤ -0.00005 | Ref (1) | **0.597** | **(0.445,0.802)** | **0.633** | **(0.459,0.874)** | **0.003** |
|  |  | -0.00005 - 0.00119 | Ref (1) | 0.947 | (0.709,1.265) | 0.796 | (0.581,1.090) | 0.154 |
|  |  | ≥ 0.00119 | Ref (1) | 0.751 | (0.564,1.000) | **0.701** | **(0.515,0.954)** | **0.025** |
| Sleep duration | | | | | | | | |
|  | Model 1 | ≤ 0.01256 | Ref (1) | **0.718** | **(0.560,0.920)** | **0.587** | **(0.444,0.774)** | **0.000** |
|  |  | 0.01256 - 0.01624 | Ref (1) | **0.752** | **(0.581,0.973)** | 0.773 | (0.591,1.010) | 0.062 |
|  |  | ≥ 0.01624 | Ref (1) | **0.540** | **(0.370,0.789)** | **0.596** | **(0.396,0.895)** | **0.009** |
|  | Model 2 | ≤ 0.01256 | Ref (1) | **0.713** | **(0.556,0.914)** | **0.626** | **(0.473,0.829)** | **0.001** |
|  |  | 0.01256 - 0.01624 | Ref (1) | 0.815 | (0.628,1.057) | 0.831 | (0.635,1.089) | 0.181 |
|  |  | ≥ 0.01624 | Ref (1) | **0.530** | **(0.358,0.783)** | **0.611** | **(0.405,0.922)** | **0.015** |
|  | Model 3 | ≤ 0.01256 | Ref (1) | **0.717** | **(0.559,0.920)** | **0.630** | **(0.476,0.835)** | **0.001** |
|  |  | 0.01256 - 0.01624 | Ref (1) | 0.812 | (0.625,1.053) | 0.832 | (0.635,1.090) | 0.185 |
|  |  | ≥ 0.01624 | Ref (1) | **0.543** | **(0.367,0.803)** | **0.631** | **(0.417,0.956)** | **0.023** |
|  | Model 4 | ≤ 0.01256 | Ref (1) | **0.740** | **(0.575,0.953)** | **0.638** | **(0.477,0.853)** | **0.002** |
|  |  | 0.01256 - 0.01624 | Ref (1) | 0.810 | (0.620,1.057) | 0.828 | (0.627,1.094) | 0.188 |
|  |  | ≥ 0.01624 | Ref (1) | **0.611** | **(0.410,0.912)** | 0.681 | (0.445,1.043) | 0.067 |
| Insomnia | |  |  |  |  |  |  |  |
|  | Model 1 | ≤ -0.00525 | Ref (1) | **0.717** | **(0.541,0.951)** | 0.769 | (0.576,1.025) | 0.081 |
|  |  | -0.00525 - 0.00268 | Ref (1) | **0.638** | **(0.484,0.840)** | **0.600** | **(0.439,0.820)** | **0.001** |
|  |  | ≥ -0.00268 | Ref (1) | **0.721** | **(0.544,0.955)** | **0.596** | **(0.438,0.810)** | **0.001** |
|  | Model 2 | ≤ -0.00525 | Ref (1) | **0.731** | **(0.549,0.972)** | 0.806 | (0.602,1.080) | 0.162 |
|  |  | -0.00525 - 0.00268 | Ref (1) | **0.647** | **(0.490,0.854)** | **0.638** | **(0.465,0.876)** | **0.003** |
|  |  | ≥ -0.00268 | Ref (1) | 0.761 | (0.573,1.010) | **0.608** | **(0.447,0.828)** | **0.002** |
|  | Model 3 | ≤ -0.00525 | Ref (1) | **0.729** | **(0.548,0.971)** | 0.817 | (0.610,1.095) | 0.194 |
|  |  | -0.00525 - 0.00268 | Ref (1) | **0.653** | **(0.494,0.863)** | **0.642** | **(0.467,0.883)** | **0.004** |
|  |  | ≥ -0.00268 | Ref (1) | **0.750** | **(0.565,0.997)** | **0.607** | **(0.446,0.827)** | **0.001** |
|  | Model 4 | ≤ -0.00525 | Ref (1) | **0.741** | **(0.552,0.994)** | 0.840 | (0.621,1.136) | 0.275 |
|  |  | -0.00525 - 0.00268 | Ref (1) | **0.684** | **(0.516,0.907)** | **0.639** | **(0.460,0.889)** | **0.005** |
|  |  | ≥ -0.00268 | Ref (1) | 0.776 | (0.582,1.035) | **0.623** | **(0.454,0.854)** | **0.003** |
| Daytime Sleepiness | | |  |  |  |  |  |  |
|  | Model 1 | ≤ 0.00587 | Ref (1) | **0.665** | **(0.506,0.873)** | **0.574** | **(0.424,0.776)** | **0.000** |
|  |  | 0.00587 - 0.00676 | Ref (1) | **0.697** | **(0.526,0.925)** | **0.736** | **(0.547,0.992)** | **0.043** |
|  |  | ≥ 0.00676 | Ref (1) | **0.711** | **(0.535,0.946)** | **0.679** | **(0.502,0.918)** | **0.011** |
|  | Model 2 | ≤ 0.00587 | Ref (1) | **0.685** | **(0.521,0.900)** | **0.639** | **(0.472,0.867)** | **0.003** |
|  |  | 0.00587 - 0.00676 | Ref (1) | **0.694** | **(0.522,0.923)** | 0.763 | (0.564,1.033) | 0.078 |
|  |  | ≥ 0.00676 | Ref (1) | **0.728** | **(0.545,0.971)** | **0.689** | **(0.508,0.935)** | **0.016** |
|  | Model 3 | ≤ 0.00587 | Ref (1) | **0.695** | **(0.528,0.913)** | **0.639** | **(0.471,0.867)** | **0.003** |
|  |  | 0.00587 - 0.00676 | Ref (1) | **0.716** | **(0.537,0.953)** | 0.767 | (0.567,1.039) | 0.086 |
|  |  | ≥ 0.00676 | Ref (1) | **0.716** | **(0.536,0.957)** | **0.689** | **(0.507,0.935)** | **0.016** |
|  | Model 4 | ≤ 0.00587 | Ref (1) | **0.663** | **(0.501,0.878)** | **0.621** | **(0.452,0.852)** | **0.002** |
|  |  | 0.00587 - 0.00676 | Ref (1) | 0.781 | (0.583,1.046) | 0.811 | (0.594,1.106) | 0.186 |
|  |  | ≥ 0.00676 | Ref (1) | 0.751 | (0.560,1.007) | **0.689** | **(0.503,0.943)** | **0.019** |
| Snoring | |  |  |  |  |  |  |  |
|  | Model 1 | ≤ 0.00020 | Ref (1) | **0.754** | **(0.573,0.992)** | 0.779 | (0.580,1.045) | 0.092 |
|  |  | 0.00020 - 0.00111 | Ref (1) | **0.707** | **(0.527,0.950)** | **0.617** | **(0.447,0.850)** | **0.003** |
|  |  | ≥ 0.00111 | Ref (1) | **0.615** | **(0.468,0.809)** | **0.588** | **(0.440,0.786)** | **0.000** |
|  | Model 2 | ≤ 0.00020 | Ref (1) | 0.793 | (0.601,1.047) | 0.849 | (0.631,1.142) | 0.267 |
|  |  | 0.00020 - 0.00111 | Ref (1) | **0.712** | **(0.529,0.957)** | **0.641** | **(0.465,0.885)** | **0.006** |
|  |  | ≥ 0.00111 | Ref (1) | **0.631** | **(0.479,0.833)** | **0.609** | **(0.454,0.818)** | **0.001** |
|  | Model 3 | ≤ 0.00020 | Ref (1) | 0.791 | (0.599,1.044) | 0.848 | (0.630,1.142) | 0.266 |
|  |  | 0.00020 - 0.00111 | Ref (1) | **0.726** | **(0.539,0.978)** | **0.645** | **(0.467,0.890)** | **0.007** |
|  |  | ≥ 0.00111 | Ref (1) | **0.620** | **(0.470,0.817)** | **0.612** | **(0.456,0.822)** | **0.001** |
|  | Model 4 | ≤ 0.00020 | Ref (1) | 0.847 | (0.639,1.122) | 0.836 | (0.614,1.140) | 0.247 |
|  |  | 0.00020 - 0.00111 | Ref (1) | **0.735** | **(0.543,0.995)** | **0.673** | **(0.485,0.934)** | **0.017** |
|  |  | ≥ 0.00111 | Ref (1) | **0.629** | **(0.473,0.835)** | **0.624** | **(0.461,0.845)** | **0.002** |

^a^Model 1 were adjusted for age, sex, race. Model 2 further adjusted for BMI, smoke, drink, sport, education, Thomson index, Family history of diabetes, Family history of hypertension, Family history of heart disease. Model 3 further adjusted for Dietary energy, Dietary fiber, Quality of diet. Model 4 further adjusted for Age at diagnosis of type 2 diabetes, Severity of diabetes.

Ref, reference; HR: hazard ratio; CI: confidence interval.

| **Table S7.** Association of sleep patterns with PAD stratified by the PRS of Chronotype, Sleep duration, Insomnia, Daytime Sleepiness and Snoring. | | | | | | | | |
| --- | --- | --- | --- | --- | --- | --- | --- | --- |
|  |  | Tertiles | Poor sleep | Intermediate sleep | | Sleep health | | P_trend_ |
|  |  |  |  | HR | 95% CI | HR | 95% CI |  |
| Chronotype | | | | | | | | |
|  | Model 1^a^ | ≤ -0.00006 | Ref (1) | 0.960 | (0.540,1.707) | 0.706 | (0.540,1.707) | 0.328 |
|  |  | -0.00006 - 0.00118 | Ref (1) | 0.607 | (0.339,1.088) | 0.541 | (0.291,1.006) | **0.049** |
|  |  | ≥ 0.00118 | Ref (1) | 0.840 | (0.504,1.400) | **0.446** | **(0.239,0.834)** | **0.011** |
|  | Model 2 | ≤ -0.00006 | Ref (1) | 1.092 | (0.610,1.954) | 0.845 | (0.426,1.679) | 0.672 |
|  |  | -0.00006 - 0.00118 | Ref (1) | 0.702 | (0.389,1.267) | 0.654 | (0.348,1.231) | 0.179 |
|  |  | ≥ 0.00118 | Ref (1) | 0.855 | (0.511,1.432) | **0.455** | **(0.242,0.854)** | **0.014** |
|  | Model 3 | ≤ -0.00006 | Ref (1) | 1.122 | (0.626,2.013) | 0.870 | (0.437,1.733) | 0.741 |
|  |  | -0.00006 - 0.00118 | Ref (1) | 0.711 | (0.393,1.288) | 0.659 | (0.350,1.242) | 0.189 |
|  |  | ≥ 0.00118 | Ref (1) | 0.853 | (0.508,1.431) | **0.445** | **(0.237,0.837)** | **0.012** |
|  | Model 4 | ≤ -0.00006 | Ref (1) | 1.169 | (0.652,2.097) | 0.728 | (0.346,1.528) | 0.490 |
|  |  | -0.00006 - 0.00118 | Ref (1) | 0.677 | (0.375,1.222) | 0.662 | (0.351,1.251) | 0.190 |
|  |  | ≥ 0.00118 | Ref (1) | 0.904 | (0.536,1.527) | **0.443** | **(0.231,0.852)** | **0.015** |
| Sleep duration | | | | | | | | |
|  | Model 1 | ≤ 0.01256 | Ref (1) | 0.833 | (0.513,1.351) | **0.437** | **(0.236,0.811)** | **0.009** |
|  |  | 0.01256 - 0.01624 | Ref (1) | 0.718 | (0.438,1.177) | 0.585 | (0.342,1.001) | 0.050 |
|  |  | ≥ 0.01624 | Ref (1) | 0.966 | (0.428,2.184) | 0.737 | (0.290,1.870) | 0.524 |
|  | Model 2 | ≤ 0.01256 | Ref (1) | 0.886 | (0.545,1.442) | **0.529** | **(0.282,0.990)** | 0.053 |
|  |  | 0.01256 - 0.01624 | Ref (1) | 0.802 | (0.487,1.322) | 0.669 | (0.389,1.151) | 0.146 |
|  |  | ≥ 0.01624 | Ref (1) | 1.025 | (0.447,2.349) | 0.781 | (0.304,2.006) | 0.612 |
|  | Model 3 | ≤ 0.01256 | Ref (1) | 0.885 | (0.543,1.442) | **0.522** | **(0.278,0.978)** | **0.048** |
|  |  | 0.01256 - 0.01624 | Ref (1) | 0.805 | (0.487,1.330) | 0.662 | (0.384,1.140) | 0.136 |
|  |  | ≥ 0.01624 | Ref (1) | 1.052 | (0.456,2.424) | 0.805 | (0.312,2.079) | 0.658 |
|  | Model 4 | ≤ 0.01256 | Ref (1) | 0.903 | (0.550,1.482) | **0.465** | **(0.239,0.906)** | **0.030** |
|  |  | 0.01256 - 0.01624 | Ref (1) | 0.807 | (0.489,1.330) | 0.639 | (0.369,1.108) | 0.110 |
|  |  | ≥ 0.01624 | Ref (1) | 1.196 | (0.515,2.781) | 0.786 | (0.284,2.174) | 0.674 |
| Insomnia | | | | | | | |  |
|  | Model 1 | ≤ -0.00526 | Ref (1) | 0.771 | (0.442,1.342) | 0.535 | (0.284,1.009) | 0.052 |
|  |  | -0.00526 - -0.00267 | Ref (1) | 0.954 | (0.550,1.655) | 0.678 | (0.351,1.310) | 0.259 |
|  |  | ≥ -0.00267 | Ref (1) | 0.681 | (0.393,1.183) | **0.453** | **(0.242,0.849)** | **0.013** |
|  | Model 2 | ≤ -0.00526 | Ref (1) | 0.822 | (0.470,1.436) | 0.594 | (0.312,1.129) | 0.112 |
|  |  | -0.00526 - -0.00267 | Ref (1) | 1.015 | (0.579,1.778) | 0.793 | (0.406,1.548) | 0.518 |
|  |  | ≥ -0.00267 | Ref (1) | 0.701 | (0.403,1.217) | **0.474** | **(0.251,0.892)** | **0.019** |
|  | Model 3 | ≤ -0.00526 | Ref (1) | 0.839 | (0.480,1.467) | 0.593 | (0.312,1.129) | 0.112 |
|  |  | -0.00526 - -0.00267 | Ref (1) | 1.034 | (0.587,1.824) | 0.777 | (0.397,1.522) | 0.484 |
|  |  | ≥ -0.00267 | Ref (1) | 0.702 | (0.403,1.222) | **0.470** | **(0.249,0.887)** | **0.019** |
|  | Model 4 | ≤ -0.00526 | Ref (1) | 0.921 | (0.523,1.623) | 0.646 | (0.336,1.240) | 0.195 |
|  |  | -0.00526 - -0.00267 | Ref (1) | 1.151 | (0.647,2.045) | 0.785 | (0.393,1.569) | 0.537 |
|  |  | ≥ -0.00267 | Ref (1) | 0.658 | (0.378,1.147) | **0.388** | **(0.197,0.765)** | **0.005** |
| Daytime Sleepiness | | | | | | | | |
|  | Model 1 | ≤ 0.00587 | Ref (1) | 1.029 | (0.582,1.820) | **0.451** | **(0.216,0.943)** | **0.039** |
|  |  | 0.00587 - 0.00676 | Ref (1) | **0.526** | **(0.315,0.881)** | **0.493** | **(0.282,0.862)** | **0.010** |
|  |  | ≥ 0.00676 | Ref (1) | 1.008 | (0.557,1.824) | 0.758 | (0.389,1.477) | 0.418 |
|  | Model 2 | ≤ 0.00587 | Ref (1) | 1.101 | (0.620,1.955) | 0.514 | (0.243,1.085) | 0.099 |
|  |  | 0.00587 - 0.00676 | Ref (1) | **0.553** | **(0.328,0.932)** | **0.543** | **(0.308,0.957)** | **0.028** |
|  |  | ≥ 0.00676 | Ref (1) | 1.103 | (0.605,2.012) | 0.846 | (0.431,1.660) | 0.638 |
|  | Model 3 | ≤ 0.00587 | Ref (1) | 1.144 | (0.642,2.038) | 0.530 | (0.250,1.123) | 0.121 |
|  |  | 0.00587 - 0.00676 | Ref (1) | **0.563** | **(0.334,0.950)** | **0.530** | **(0.300,0.934)** | **0.023** |
|  |  | ≥ 0.00676 | Ref (1) | 1.081 | (0.591,1.977) | 0.826 | (0.420,1.626) | 0.588 |
|  | Model 4 | ≤ 0.00587 | Ref (1) | 1.137 | (0.638,2.025) | **0.435** | **(0.195,0.969)** | **0.060** |
|  |  | 0.00587 - 0.00676 | Ref (1) | 0.620 | (0.365,1.051) | **0.495** | **(0.275,0.889)** | **0.016** |
|  |  | ≥ 0.00676 | Ref (1) | 1.132 | (0.617,2.077) | 0.845 | (0.422,1.694) | 0.658 |
| Snoring | | | | | | | | |
|  | Model 1 | ≤ 0.00020 | Ref (1) | 1.302 | (0.707,2.397) | 0.888 | (0.444,1.778) | 0.721 |
|  |  | 0.00020 - 0.00111 | Ref (1) | 0.786 | (0.467,1.324) | **0.278** | **(0.131,0.594)** | **0.001** |
|  |  | ≥ 0.00111 | Ref (1) | **0.526** | **(0.302,0.918)** | 0.619 | (0.354,1.082) | 0.086 |
|  | Model 2 | ≤ 0.00020 | Ref (1) | 1.433 | (0.771,2.665) | 1.010 | (0.500,2.038) | 0.988 |
|  |  | 0.00020 - 0.00111 | Ref (1) | 0.827 | (0.489,1.397) | **0.313** | **(0.146,0.671)** | **0.003** |
|  |  | ≥ 0.00111 | Ref (1) | 0.601 | (0.343,1.052) | 0.739 | (0.418,1.306) | 0.273 |
|  | Model 3 | ≤ 0.00020 | Ref (1) | 1.475 | (0.792,2.747) | 1.050 | (0.519,2.123) | 0.897 |
|  |  | 0.00020 - 0.00111 | Ref (1) | 0.872 | (0.513,1.483) | **0.312** | **(0.145,0.670)** | **0.003** |
|  |  | ≥ 0.00111 | Ref (1) | 0.603 | (0.344,1.057) | 0.729 | (0.412,1.291) | 0.257 |
|  | Model 4 | ≤ 0.00020 | Ref (1) | 1.497 | (0.803,2.789) | 1.082 | (0.535,2.190) | 0.827 |
|  |  | 0.00020 - 0.00111 | Ref (1) | 0.904 | (0.526,1.556) | **0.300** | **(0.134,0.671)** | **0.004** |
|  |  | ≥ 0.00111 | Ref (1) | 0.657 | (0.374,1.155) | 0.648 | (0.356,1.179) | 0.140 |

^a^Model 1 were adjusted for age, sex, race. Model 2 further adjusted for BMI, smoke, drink, sport, education, Thomson index, Family history of diabetes, Family history of hypertension, Family history of heart disease. Model 3 further adjusted for Dietary energy, Dietary fiber, Quality of diet. Model 4 further adjusted for Age at diagnosis of type 2 diabetes, Severity of diabetes.

Ref, reference; HR: hazard ratio; CI: confidence interval.

| **Table S8.** Association of sleep patterns with Heart Failure stratified by the PRS Of Chronotype, Sleep duration, Insomnia, Daytime Sleepiness and Snoring. | | | | | | | | |
| --- | --- | --- | --- | --- | --- | --- | --- | --- |
|  |  | Tertiles | Poor sleep | Intermediate sleep | | Sleep health | | P_trend_ |
|  |  |  |  | HR | 95% CI | HR | 95% CI |  |
| Chronotype | | | | | | | | |
|  | Model 1^a^ | ≤ -0.00006 | Ref (1) | 0.693 | (0.447,1.076) | 0.668 | (0.412,1.084) | 0.086 |
|  |  | -0.00006 - 0.00118 | Ref (1) | **0.479** | **(0.292,0.787)** | **0.478** | **(0.285,0.802)** | **0.003** |
|  |  | ≥ 0.00118 | Ref (1) | 0.653 | (0.418,1.021) | **0.557** | **(0.339,0.915)** | **0.019** |
|  | Model 2 | ≤ -0.00006 | Ref (1) | 0.729 | (0.468,1.136) | 0.705 | (0.433,1.148) | 0.140 |
|  |  | -0.00006 - 0.00118 | Ref (1) | **0.498** | **(0.302,0.821)** | **0.521** | **(0.308,0.882)** | **0.010** |
|  |  | ≥ 0.00118 | Ref (1) | 0.673 | (0.429,1.056) | 0.613 | (0.371,1.013) | 0.052 |
|  | Model 3 | ≤ -0.00006 | Ref (1) | 0.730 | (0.468,1.140) | 0.708 | (0.435,1.154) | 0.146 |
|  |  | -0.00006 - 0.00118 | Ref (1) | **0.501** | **(0.304,0.827)** | **0.523** | **(0.309,0.886)** | **0.011** |
|  |  | ≥ 0.00118 | Ref (1) | 0.682 | (0.434,1.071) | 0.612 | (0.370,1.012) | 0.051 |
|  | Model 4 | ≤ -0.00006 | Ref (1) | 0.745 | (0.474,1.169) | 0.725 | (0.441,1.193) | 0.182 |
|  |  | -0.00006 - 0.00118 | Ref (1) | **0.531** | **(0.320,0.880)** | **0.506** | **(0.292,0.876)** | **0.010** |
|  |  | ≥ 0.00118 | Ref (1) | 0.716 | (0.452,1.134) | 0.631 | (0.376,1.059) | 0.076 |
| Sleep duration | | | | | | | | |
|  | Model 1 | ≤ 0.01256 | Ref (1) | **0.568** | **(0.375,0.861)** | **0.512** | **(0.325,0.809)** | **0.003** |
|  |  | 0.01256 - 0.01624 | Ref (1) | **0.564** | **(0.371,0.856)** | **0.518** | **(0.330,0.813)** | **0.003** |
|  |  | ≥ 0.01624 | Ref (1) | 0.849 | (0.459,1.571) | 0.856 | (0.440,1.665) | 0.649 |
|  | Model 2 | ≤ 0.01256 | Ref (1) | **0.570** | **(0.375,0.865)** | **0.548** | **(0.345,0.869)** | **0.007** |
|  |  | 0.01256 - 0.01624 | Ref (1) | **0.623** | **(0.409,0.950)** | **0.594** | **(0.377,0.937)** | **0.019** |
|  |  | ≥ 0.01624 | Ref (1) | 0.851 | (0.452,1.602) | 0.873 | (0.443,1.718) | 0.701 |
|  | Model 3 | ≤ 0.01256 | Ref (1) | **0.567** | **(0.373,0.861)** | **0.542** | **(0.341,0.861)** | **0.006** |
|  |  | 0.01256 - 0.01624 | Ref (1) | **0.626** | **(0.410,0.955)** | **0.603** | **(0.381,0.952)** | **0.023** |
|  |  | ≥ 0.01624 | Ref (1) | 0.811 | (0.428,1.535) | 0.833 | (0.421,1.646) | 0.611 |
|  | Model 4 | ≤ 0.01256 | Ref (1) | **0.575** | **(0.376,0.881)** | **0.535** | **(0.331,0.864)** | **0.007** |
|  |  | 0.01256 - 0.01624 | Ref (1) | **0.629** | **(0.411,0.962)** | **0.594** | **(0.374,0.945)** | **0.022** |
|  |  | ≥ 0.01624 | Ref (1) | 0.893 | (0.472,1.691) | 0.830 | (0.413,1.667) | 0.601 |
| Insomnia | | | | | | | | |
|  | Model 1 | ≤ -0.00525 | Ref (1) | **0.595** | **(0.375,0.943)** | **0.477** | **(0.285,0.797)** | **0.004** |
|  |  | -0.00525 - -0.00268 | Ref (1) | **0.555** | **(0.360,0.854)** | **0.613** | **(0.383,0.981)** | **0.026** |
|  |  | ≥ -0.00268 | Ref (1) | 0.720 | (0.444,1.170) | 0.611 | (0.365,1.024) | **0.060** |
|  | Model 2 | ≤ -0.00525 | Ref (1) | **0.614** | **(0.386,0.977)** | **0.497** | **(0.294,0.838)** | **0.007** |
|  |  | -0.00525 - -0.00268 | Ref (1) | **0.605** | **(0.391,0.936)** | 0.692 | (0.429,1.115) | 0.092 |
|  |  | ≥ -0.00268 | Ref (1) | 0.749 | (0.461,1.218) | 0.654 | (0.389,1.099) | 0.106 |
|  | Model 3 | ≤ -0.00525 | Ref (1) | **0.593** | **(0.372,0.946)** | **0.489** | **(0.289,0.828)** | **0.006** |
|  |  | -0.00525 - -0.00268 | Ref (1) | **0.610** | **(0.393,0.945)** | 0.690 | (0.427,1.113) | 0.092 |
|  |  | ≥ -0.00268 | Ref (1) | 0.763 | (0.469,1.242) | 0.663 | (0.395,1.115) | 0.119 |
|  | Model 4 | ≤ -0.00525 | Ref (1) | **0.621** | **(0.388,0.994)** | **0.518** | **(0.305,0.879)** | **0.012** |
|  |  | -0.00525 - -0.00268 | Ref (1) | 0.697 | (0.446,1.089) | 0.723 | (0.439,1.190) | 0.164 |
|  |  | ≥ -0.00268 | Ref (1) | 0.761 | (0.466,1.243) | 0.659 | (0.386,1.125) | 0.121 |
| Daytime Sleepiness | | | | | | | | |
|  | Model 1 | ≤ 0.00587 | Ref (1) | **0.599** | **(0.376,0.953)** | **0.458** | **(0.269,0.781)** | **0.003** |
|  |  | 0.00587 - 0.00676 | Ref (1) | 0.690 | (0.442,1.077) | 0.649 | (0.399,1.056) | 0.074 |
|  |  | ≥ 0.00676 | Ref (1) | **0.556** | **(0.349,0.885)** | **0.588** | **(0.364,0.951)** | **0.024** |
|  | Model 2 | ≤ 0.00587 | Ref (1) | 0.629 | (0.394,1.005) | **0.523** | **(0.304,0.902)** | **0.015** |
|  |  | 0.00587 - 0.00676 | Ref (1) | 0.749 | (0.478,1.175) | 0.758 | (0.463,1.243) | 0.250 |
|  |  | ≥ 0.00676 | Ref (1) | **0.595** | **(0.372,0.951)** | **0.611** | **(0.376,0.993)** | **0.039** |
|  | Model 3 | ≤ 0.00587 | Ref (1) | 0.632 | (0.395,1.010) | **0.520** | **(0.301,0.898)** | **0.015** |
|  |  | 0.00587 - 0.00676 | Ref (1) | 0.749 | (0.476,1.176) | 0.760 | (0.464,1.246) | 0.254 |
|  |  | ≥ 0.00676 | Ref (1) | **0.597** | **(0.373,0.955)** | 0.618 | (0.380,1.007) | **0.044** |
|  | Model 4 | ≤ 0.00587 | Ref (1) | 0.624 | (0.389,1.001) | **0.512** | **(0.293,0.895)** | **0.014** |
|  |  | 0.00587 - 0.00676 | Ref (1) | 0.797 | (0.506,1.256) | 0.721 | (0.437,1.189) | 0.191 |
|  |  | ≥ 0.00676 | Ref (1) | 0.681 | (0.421,1.102) | 0.644 | (0.387,1.072) | 0.081 |
| Snoring | | | | | | | | |
|  | Model 1 | ≤ 0.00020 | Ref (1) | 0.658 | (0.411,1.052) | 0.648 | (0.389,1.078) | 0.082 |
|  |  | 0.00020 - 0.00111 | Ref (1) | 0.876 | (0.554,1.387) | **0.561** | **(0.327,0.963)** | **0.037** |
|  |  | ≥ 0.00111 | Ref (1) | **0.404** | **(0.255,0.639)** | **0.499** | **(0.316,0.788)** | **0.002** |
|  | Model 2 | ≤ 0.00020 | Ref (1) | 0.704 | (0.439,1.130) | 0.706 | (0.423,1.181) | 0.164 |
|  |  | 0.00020 - 0.00111 | Ref (1) | 0.912 | (0.574,1.448) | 0.613 | (0.355,1.059) | 0.084 |
|  |  | ≥ 0.00111 | Ref (1) | **0.433** | **(0.272,0.689)** | **0.547** | **(0.343,0.874)** | **0.007** |
|  | Model 3 | ≤ 0.00020 | Ref (1) | 0.703 | (0.438,1.130) | 0.706 | (0.422,1.181) | 0.164 |
|  |  | 0.00020 - 0.00111 | Ref (1) | 0.945 | (0.593,1.505) | 0.615 | (0.355,1.063) | 0.087 |
|  |  | ≥ 0.00111 | Ref (1) | **0.439** | **(0.276,0.699)** | **0.569** | **(0.356,0.909)** | **0.010** |
|  | Model 4 | ≤ 0.00020 | Ref (1) | 0.711 | (0.443,1.141) | 0.693 | (0.412,1.168) | 0.148 |
|  |  | 0.00020 - 0.00111 | Ref (1) | 1.042 | (0.645,1.684) | 0.676 | (0.386,1.185) | 0.185 |
|  |  | ≥ 0.00111 | Ref (1) | **0.477** | **(0.298,0.765)** | **0.564** | **(0.345,0.921)** | **0.013** |

^a^Model 1 were adjusted for age, sex, race. Model 2 further adjusted for BMI, smoke, drink, sport, education, Thomson index, Family history of diabetes, Family history of hypertension, Family history of heart disease. Model 3 further adjusted for Dietary energy, Dietary fiber, Quality of diet. Model 4 further adjusted for Age at diagnosis of type 2 diabetes, Severity of diabetes.

Ref, reference; HR: hazard ratio; CI: confidence interval.

| **Table S9.** Associations between sleep patterns and CVD events after excluding participants with Sleep Disorders | | | | | | | |
| --- | --- | --- | --- | --- | --- | --- | --- |
|  |  | Poor sleep | Intermediate sleep | | Sleep health | | P_trend_ |
|  |  |  | HR | 95% CI | HR | 95% CI |  |
| **CVD Mortality** | | | | | | | |
|  | Model 1^a^ | Ref (1) | 0.798 | (0.618,1.030) | **0.546** | **(0.404,0.739)** | **0.000** |
|  | Model 2 | Ref (1) | 0.850 | (0.658,1.099) | **0.599** | **(0.442,0.811)** | **0.001** |
|  | Model 3 | Ref (1) | 0.855 | (0.661,1.106) | **0.601** | **(0.443,0.815)** | **0.001** |
|  | Model 4 | Ref (1) | 0.876 | (0.674,1.140) | **0.632** | **(0.463,0.862)** | **0.004** |
| **ASCVD** | | | | | | | |
|  | Model 1 | Ref (1) | **0.783** | **(0.677,0.907)** | **0.746** | **(0.637,0.873)** | **0.000** |
|  | Model 2 | Ref (1) | **0.826** | **(0.713,0.957)** | **0.807** | **(0.689,0.946)** | **0.007** |
|  | Model 3 | Ref (1) | **0.827** | **(0.714,0.958)** | **0.809** | **(0.690,0.948)** | **0.008** |
|  | Model 4 | Ref (1) | 0.867 | (0.745,1.008) | **0.846** | **(0.718,0.996)** | **0.043** |
| **CAD** | | | | | | | |
|  | Model 1 | Ref (1) | **0.731** | **(0.619,0.865)** | **0.705** | **(0.589,0.843)** | **0.000** |
|  | Model 2 | Ref (1) | **0.759** | **(0.642,0.898)** | **0.742** | **(0.619,0.889)** | **0.001** |
|  | Model 3 | Ref (1) | **0.761** | **(0.643,0.900)** | **0.746** | **(0.623,0.894)** | **0.001** |
|  | Model 4 | Ref (1) | **0.801** | **(0.674,0.951)** | **0.755** | **(0.626,0.911)** | **0.003** |
| **PAD** | | | | | | | |
|  | Model 1 | Ref (1) | 0.873 | (0.625,1.218) | **0.562** | **(0.378,0.833)** | **0.004** |
|  | Model 2 | Ref (1) | 0.931 | (0.666,1.302) | **0.620** | **(0.416,0.922)** | **0.020** |
|  | Model 3 | Ref (1) | 0.949 | (0.678,1.329) | **0.623** | **(0.419,0.928)** | **0.023** |
|  | Model 4 | Ref (1) | 1.004 | (0.713,1.413) | **0.616** | **(0.408,0.930)** | **0.026** |
| **HF** | | | | | | | |
|  | Model 1 | Ref (1) | **0.694** | **(0.528,0.913)** | **0.565** | **(0.415,0.769)** | **0.000** |
|  | Model 2 | Ref (1) | **0.729** | **(0.554,0.959)** | **0.601** | **(0.440,0.820)** | **0.001** |
|  | Model 3 | Ref (1) | **0.731** | **(0.555,0.962)** | **0.603** | **(0.442,0.824)** | **0.001** |
|  | Model 4 | Ref (1) | 0.772 | (0.584,1.022) | **0.623** | **(0.452,0.858)** | **0.003** |

^a^Model 1 were adjusted for age, sex, race. Model 2 further adjusted for BMI, smoke, drink, sport, education, Thomson index, Family history of diabetes, Family history of hypertension, Family history of heart disease. Model 3 further adjusted for Dietary energy, Dietary fiber, Quality of diet. Model 4 further adjusted for Age at diagnosis of type 2 diabetes, Severity of diabetes.

CVD: cardiovascular disease; ASCVD: atherosclerosis cardiovascular disease; CAD: coronary artery disease PAD: peripheral arterial disease; HF: Heart Failure; Ref, reference; HR: hazard ratio; CI: confidence interval.

| **Table S10.** Associations between sleep patterns and CVD events after excluding participants with Depression | | | | | | | |
| --- | --- | --- | --- | --- | --- | --- | --- |
|  |  | Poor sleep | Intermediate sleep | | Sleep health | | P_trend_ |
|  |  |  | HR | 95% CI | HR | 95% CI |  |
| **CVD Mortality** | | | | | | | |
|  | Model 1^a^ | Ref (1) | 0.797 | (0.615,1.033) | **0.583** | **(0.433,0.785)** | **0.000** |
|  | Model 2 | Ref (1) | 0.837 | (0.645,1.086) | **0.628** | **(0.465,0.847)** | **0.002** |
|  | Model 3 | Ref (1) | 0.842 | (0.649,1.093) | **0.629** | **(0.466,0.849)** | **0.002** |
|  | Model 4 | Ref (1) | 0.852 | (0.654,1.110) | **0.651** | **(0.480,0.884)** | **0.006** |
| **ASCVD** | | | | | | | |
|  | Model 1 | Ref (1) | **0.782** | **(0.672,0.909)** | **0.736** | **(0.626,0.865)** | **0.000** |
|  | Model 2 | Ref (1) | **0.816** | **(0.702,0.950)** | **0.788** | **(0.670,0.928)** | **0.004** |
|  | Model 3 | Ref (1) | **0.816** | **(0.702,0.949)** | **0.790** | **(0.671,0.929)** | **0.004** |
|  | Model 4 | Ref (1) | **0.850** | **(0.728,0.993)** | **0.817** | **(0.691,0.967)** | **0.018** |
| **CAD** | | | | | | | |
|  | Model 1 | Ref (1) | **0.761** | **(0.640,0.904)** | **0.742** | **(0.618,0.891)** | **0.001** |
|  | Model 2 | Ref (1) | **0.774** | **(0.651,0.920)** | **0.766** | **(0.637,0.920)** | **0.005** |
|  | Model 3 | Ref (1) | **0.773** | **(0.650,0.919)** | **0.768** | **(0.639,0.923)** | **0.005** |
|  | Model 4 | Ref (1) | **0.797** | **(0.668,0.951)** | **0.764** | **(0.632,0.924)** | **0.005** |
| **PAD** | | | | | | | |
|  | Model 1 | Ref (1) | 0.789 | (0.563,1.105) | **0.548** | **(0.372,0.810)** | **0.002** |
|  | Model 2 | Ref (1) | 0.835 | (0.595,1.173) | **0.601** | **(0.406,0.890)** | **0.011** |
|  | Model 3 | Ref (1) | 0.849 | (0.604,1.193) | **0.606** | **(0.409,0.897)** | **0.013** |
|  | Model 4 | Ref (1) | 0.889 | (0.631,1.253) | **0.583** | **(0.388,0.876)** | **0.010** |
| **HF** | | | | | | | |
|  | Model 1 | Ref (1) | **0.697** | **(0.527,0.920)** | **0.613** | **(0.452,0.831)** | **0.001** |
|  | Model 2 | Ref (1) | **0.723** | **(0.547,0.957)** | **0.648** | **(0.477,0.882)** | **0.005** |
|  | Model 3 | Ref (1) | **0.722** | **(0.546,0.956)** | **0.648** | **(0.477,0.882)** | **0.005** |
|  | Model 4 | Ref (1) | **0.747** | **(0.562,0.991)** | **0.649** | **(0.473,0.890)** | **0.006** |

^a^Model 1 were adjusted for age, sex, race. Model 2 further adjusted for BMI, smoke, drink, sport, education, Thomson index, Family history of diabetes, Family history of hypertension, Family history of heart disease. Model 3 further adjusted for Dietary energy, Dietary fiber, Quality of diet. Model 4 further adjusted for Age at diagnosis of type 2 diabetes, Severity of diabetes.

CVD: cardiovascular disease; ASCVD: atherosclerosis cardiovascular disease; CAD: coronary artery disease PAD: peripheral arterial disease; HF: Heart Failure; Ref, reference; HR: hazard ratio; CI: confidence interval.

| **Table S11.** Associations between sleep patterns and CVD events after excluding participants on Shift | | | | | | | |
| --- | --- | --- | --- | --- | --- | --- | --- |
|  |  | Poor sleep | Intermediate sleep | | Sleep health | | P_trend_ |
|  |  |  | HR | 95% CI | HR | 95% CI |  |
| **CVD Mortality** | | | | | | | |
|  | Model 1^a^ | Ref (1) | **0.705** | **(0.544,0.912）** | **0.545** | **(0.405,0.733)** | **0.000** |
|  | Model 2 | Ref (1) | 0.778 | (0.601,1.009） | **0.624** | **(0.462,0.842)** | **0.002** |
|  | Model 3 | Ref (1) | 0.786 | (0.606,1.019） | **0.628** | **(0.465,0.847)** | **0.002** |
|  | Model 4 | Ref (1) | 0.794 | (0.609,1.035） | **0.665** | **(0.489,0.905)** | **0.008** |
| **AS** | | | | | | | |
|  | Model 1 | Ref (1) | **0.731** | **(0.628,0.850）** | **0.655** | **(0.554,0.775)** | **0.000** |
|  | Model 2 | Ref (1) | **0.773** | **(0.664,0.900）** | **0.722** | **(0.610,0.855)** | **0.000** |
|  | Model 3 | Ref (1) | **0.773** | **(0.664,0.901）** | **0.722** | **(0.610,0.855)** | **0.000** |
|  | Model 4 | Ref (1) | **0.809** | **(0.691,0.946）** | **0.752** | **(0.631,0.896)** | **0.001** |
| **CAD** | | | | | | | |
|  | Model 1 | Ref (1) | **0.676** | **(0.566,0.807）** | **0.655** | **(0.542,0.793)** | **0.000** |
|  | Model 2 | Ref (1) | **0.702** | **(0.588,0.839）** | **0.698** | **(0.576,0.845)** | **0.000** |
|  | Model 3 | Ref (1) | **0.703** | **(0.588,0.840）** | **0.700** | **(0.578,0.848)** | **0.000** |
|  | Model 4 | Ref (1) | **0.734** | **(0.612,0.881）** | **0.713** | **(0.584,0.870)** | **0.001** |
| **PAD** | | | | | | | |
|  | Model 1 | Ref (1) | 0.704 | (0.492,1.007） | **0.511** | **(0.337,0.776)** | **0.001** |
|  | Model 2 | Ref (1) | 0.758 | (0.528,1.087） | **0.572** | **(0.376,0.872)** | **0.009** |
|  | Model 3 | Ref (1) | 0.765 | (0.533,1.098） | **0.577** | **(0.379,0.879)** | **0.010** |
|  | Model 4 | Ref (1) | 0.783 | (0.543,1.129） | **0.567** | **(0.366,0.878)** | **0.010** |
| **HF** | | | | | | | |
|  | Model 1 | Ref (1) | **0.653** | **(0.495,0.863）** | **0.531** | **(0.387,0.729)** | **0.000** |
|  | Model 2 | Ref (1) | **0.696** | **(0.526,0.920）** | **0.584** | **(0.424,0.804)** | **0.001** |
|  | Model 3 | Ref (1) | **0.696** | **(0.526,0.921）** | **0.585** | **(0.424,0.805)** | **0.001** |
|  | Model 4 | Ref (1) | **0.737** | **(0.554,0.981）** | **0.623** | **(0.448,0.867)** | **0.004** |

^a^Model 1 were adjusted for age, sex, race. Model 2 further adjusted for BMI, smoke, drink, sport, education, Thomson index, Family history of diabetes, Family history of hypertension, Family history of heart disease. Model 3 further adjusted for Dietary energy, Dietary fiber, Quality of diet. Model 4 further adjusted for Age at diagnosis of type 2 diabetes, Severity of diabetes.

CVD: cardiovascular disease; ASCVD: atherosclerosis cardiovascular disease; CAD: coronary artery disease PAD: peripheral arterial disease; HF: Heart Failure; Ref, reference; HR: hazard ratio; CI: confidence interval.

| **Table S12.** Associations between sleep patterns and CVD events after excluding participants with Severe Diabetes | | | | | | | |
| --- | --- | --- | --- | --- | --- | --- | --- |
|  |  | Poor sleep | Intermediate sleep | | Sleep health | | P_trend_ |
|  |  |  | HR | 95% CI | HR | 95% CI |  |
| **CVD Mortality** | | | | | | | |
|  | Model 1^a^ | Ref (1) | **0.770** | **(0.601,0.986)** | **0.584** | **(0.440,0.775)** | **0.000** |
|  | Model 2 | Ref (1) | 0.831 | (0.648,1.066) | **0.654** | **(0.491,0.870)** | **0.003** |
|  | Model 3 | Ref (1) | 0.837 | (0.652,1.074) | **0.657** | **(0.494,0.875)** | **0.004** |
|  | Model 4 | Ref (1) | 0.857 | (0.668,1.100) | **0.687** | **(0.516,0.915)** | **0.010** |
| **AS** | | | | | | | |
|  | Model 1 | Ref (1) | **0.764** | **(0.662,0.882)** | **0.702** | **(0.601,0.819)** | **0.000** |
|  | Model 2 | Ref (1) | **0.812** | **(0.703,0.938)** | **0.777** | **(0.664,0.909)** | **0.001** |
|  | Model 3 | Ref (1) | **0.815** | **(0.705,0.941)** | **0.780** | **(0.667,0.913)** | **0.002** |
|  | Model 4 | Ref (1) | **0.829** | **(0.718,0.958)** | **0.801** | **(0.684,0.937)** | **0.005** |
| **CAD** | | | | | | | |
|  | Model 1 | Ref (1) | **0.730** | **(0.619,0.861)** | **0.673** | **(0.563,0.804)** | **0.000** |
|  | Model 2 | Ref (1) | **0.758** | **(0.642,0.894)** | **0.716** | **(0.599,0.857)** | **0.000** |
|  | Model 3 | Ref (1) | **0.761** | **(0.645,0.898)** | **0.722** | **(0.604,0.864)** | **0.000** |
|  | Model 4 | Ref (1) | **0.774** | **(0.655,0.913)** | **0.748** | **(0.625,0.895)** | **0.001** |
| **PAD** | | | | | | | |
|  | Model 1 | Ref (1) | 0.791 | (0.572,1.094) | **0.533** | **(0.365,0.779)** | **0.001** |
|  | Model 2 | Ref (1) | 0.860 | (0.621,1.192) | **0.610** | **(0.416,0.894)** | **0.012** |
|  | Model 3 | Ref (1) | 0.875 | (0.631,1.214) | **0.612** | **(0.417,0.898)** | **0.013** |
|  | Model 4 | Ref (1) | 0.902 | (0.650,1.252) | **0.629** | **(0.428,0.925)** | **0.020** |
| **HF** | | | | | | | |
|  | Model 1 | Ref (1) | **0.695** | **(0.532,0.907)** | **0.598** | **(0.445,0.803)** | **0.000** |
|  | Model 2 | Ref (1) | **0.736** | **(0.563,0.962)** | **0.652** | **(0.485,0.878)** | **0.004** |
|  | Model 3 | Ref (1) | **0.736** | **(0.563,0.963)** | **0.654** | **(0.486,0.881)** | **0.004** |
|  | Model 4 | Ref (1) | **0.748** | **(0.572,0.978)** | **0.676** | **(0.501,0.910)** | **0.008** |

^a^Model 1 were adjusted for age, sex, race. Model 2 further adjusted for BMI, smoke, drink, sport, education, Thomson index, Family history of diabetes, Family history of hypertension, Family history of heart disease. Model 3 further adjusted for Dietary energy, Dietary fiber, Quality of diet. Model 4 further adjusted for Age at diagnosis of type 2 diabetes, Severity of diabetes.

CVD: cardiovascular disease; ASCVD: atherosclerosis cardiovascular disease; CAD: coronary artery disease PAD: peripheral arterial disease; HF: Heart Failure; Ref, reference; HR: hazard ratio; CI: confidence interval.

| **Table S13.** Stratified analysis of the association between sleep patterns and CVD Mortality | | | | | | | | |
| --- | --- | --- | --- | --- | --- | --- | --- | --- |
|  | | Poor sleep | | Intermediate sleep | | Sleep health | | P_trend_ |
|  |  |  |  | HR | 95% CI | HR | 95% CI |  |
| Age | | | | | | | | |
|  | Model 1^a^ | ≤ 60 | Ref (1) | 0.705 | (0.458,1.085) | **0.592** | **(0.366,0.959)** | **0.029** |
|  |  | > 60 | Ref (1) | 0.816 | (0.608,1.095) | **0.575** | **(0.408,0.810)** | **0.002** |
|  | Model 2 | ≤ 60 | Ref (1) | 0.752 | (0.487,1.162) | 0.668 | (0.411,1.086) | **0.093** |
|  |  | > 60 | Ref (1) | 0.875 | (0.651,1.177) | **0.633** | **(0.448,0.894)** | **0.010** |
|  | Model 3 | ≤ 60 | Ref (1) | 0.744 | (0.481,1.151) | 0.654 | (0.402,1.064) | **0.079** |
|  |  | > 60 | Ref (1) | 0.883 | (0.656,1.188) | **0.641** | **(0.453,0.906)** | **0.012** |
|  | Model 4 | ≤ 60 | Ref (1) | 0.801 | (0.512,1.253) | 0.723 | (0.438,1.195) | **0.194** |
|  |  | > 60 | Ref (1) | 0.872 | (0.647,1.175) | **0.636** | **(0.448,0.904)** | **0.012** |
| Sex | | | | | | | | |
|  | Model 1 | Female | Ref (1) | 0.790 | (0.484,1.291) | 0.536 | (0.277,1.038) | 0.060 |
|  |  | Male | Ref (1) | 0.777 | (0.588,1.027) | **0.595** | **(0.436,0.811)** | **0.001** |
|  | Model 2 | Female | Ref (1) | 0.808 | (0.492,1.326) | 0.592 | (0.305,1.149) | 0.113 |
|  |  | Male | Ref (1) | 0.834 | (0.63,1.104) | **0.662** | **(0.484,0.905)** | **0.010** |
|  | Model 3 | Female | Ref (1) | 0.807 | (0.492,1.324) | 0.593 | (0.305,1.154) | 0.115 |
|  |  | Male | Ref (1) | 0.835 | (0.63,1.107) | **0.664** | **(0.485,0.908)** | **0.010** |
|  | Model 4 | Female | Ref (1) | 0.801 | (0.484,1.326) | 0.611 | (0.313,1.194) | 0.136 |
|  |  | Male | Ref (1) | 0.866 | (0.651,1.152) | **0.690** | **(0.501,0.951)** | **0.023** |
| Education | | | | | | | | |
|  | Model 1 | < High school | Ref (1) | 0.738 | (0.501,1.086) | **0.378** | **(0.228,0.629)** | **0.000** |
|  |  | $\geq$High school | Ref (1) | 0.818 | (0.599,1.118) | 0.737 | (0.524,1.035) | 0.077 |
|  | Model 2 | < High school | Ref (1) | 0.809 | (0.548,1.196) | **0.435** | **(0.260,0.728)** | **0.002** |
|  |  | $\geq$High school | Ref (1) | 0.882 | (0.644,1.207) | 0.804 | (0.571,1.132) | 0.210 |
|  | Model 3 | < High school | Ref (1) | 0.815 | (0.551,1.206) | **0.441** | **(0.263,0.739)** | **0.002** |
|  |  | $\geq$High school | Ref (1) | 0.884 | (0.645,1.211) | 0.808 | (0.573,1.139) | 0.221 |
|  | Model 4 | < High school | Ref (1) | 0.782 | (0.525,1.166) | **0.420** | **(0.245,0.720)** | **0.002** |
|  |  | $\geq$High school | Ref (1) | 0.912 | (0.664,1.252) | 0.842 | (0.595,1.193) | 0.332 |
| Townsend deprivation index | | | | | | | | |
|  | Model 1 | ≤ -3.11 | Ref (1) | 0.861 | (0.505,1.467) | 1.009 | (0.589,1.729) | 0.917 |
|  |  | -3.11 - -0.52 | Ref (1) | 0.873 | (0.579,1.316) | **0.456** | **(0.274,0.760)** | **0.003** |
|  |  | ≥ -0.52 | Ref (1) | 0.737 | (0.510,1.067) | **0.534** | **(0.339,0.840)** | **0.005** |
|  | Model 2 | ≤ -3.11 | Ref (1) | 0.846 | (0.495,1.445) | 0.987 | (0.574,1.695) | 0.976 |
|  |  | -3.11 - -0.52 | Ref (1) | 0.903 | (0.598,1.363) | **0.490** | **(0.293,0.820)** | **0.008** |
|  |  | ≥ -0.52 | Ref (1) | 0.816 | (0.563,1.184) | **0.584** | **(0.370,0.922)** | **0.020** |
|  | Model 3 | ≤ -3.11 | Ref (1) | 0.843 | (0.493,1.440) | 0.988 | (0.575,1.698) | 0.971 |
|  |  | -3.11 - -0.52 | Ref (1) | 0.922 | (0.610,1.394) | **0.503** | **(0.300,0.842)** | **0.011** |
|  |  | ≥ -0.52 | Ref (1) | 0.818 | (0.563,1.189) | **0.59** | **(0.373,0.932)** | **0.023** |
|  | Model 4 | ≤ -3.11 | Ref (1) | 0.839 | (0.489,1.438) | 0.932 | (0.533,1.630) | 0.853 |
|  |  | -3.11 - -0.52 | Ref (1) | 0.964 | (0.634,1.464) | **0.542** | **(0.322,0.911)** | **0.026** |
|  |  | ≥ -0.52 | Ref (1) | 0.820 | (0.560,1.200) | 0.629 | (0.394,1.005) | **0.049** |
| Age at diagnosis of type 2 diabetes | | | | | | | | |
|  | Model 1 | ≤ 54 | Ref (1) | 0.764 | (0.462,1.263) | 0.632 | (0.363,1.098) | 0.099 |
|  |  | 54 - 61 | Ref (1) | 0.740 | (0.488,1.123) | **0.506** | **(0.307,0.835)** | **0.007** |
|  |  | ≥ 62 | Ref (1) | 0.818 | (0.571,1.174) | **0.622** | **(0.412,0.940)** | **0.024** |
|  | Model 2 | ≤ 54 | Ref (1) | 0.777 | (0.468,1.288) | 0.713 | (0.406,1.254) | 0.224 |
|  |  | 54 - 61 | Ref (1) | 0.765 | (0.503,1.164) | **0.548** | **(0.330,0.908)** | **0.018** |
|  |  | ≥ 62 | Ref (1) | 0.885 | (0.615,1.273) | 0.680 | (0.448,1.032) | 0.071 |
|  | Model 3 | ≤ 54 | Ref (1) | 0.791 | (0.476,1.314) | 0.722 | (0.410,1.269) | 0.243 |
|  |  | 54 - 61 | Ref (1) | 0.777 | (0.509,1.185) | **0.553** | **(0.333,0.918)** | **0.021** |
|  |  | ≥ 62 | Ref (1) | 0.892 | (0.620,1.284) | 0.688 | (0.454,1.045) | 0.081 |
|  | Model 4 | ≤ 54 | Ref (1) | 0.863 | (0.511,1.458) | 0.805 | (0.447,1.451) | 0.461 |
|  |  | 54 - 61 | Ref (1) | 0.742 | (0.482,1.142) | **0.539** | **(0.320,0.909)** | **0.018** |
|  |  | ≥ 62 | Ref (1) | 0.890 | (0.616,1.284) | 0.697 | (0.459,1.060) | 0.093 |
| Severity of type 2 diabetes | | | | | | | | |
|  | Model 1 | 1 | Ref (1) | 0.813 | (0.531,1.247) | 0.617 | (0.379,1.006) | 0.052 |
|  |  | 2 | Ref (1) | 0.755 | (0.522,1.092) | **0.591** | **(0.388,0.900)** | **0.013** |
|  |  | 3 | Ref (1) | 0.895 | (0.541,1.481) | 0.635 | (0.349,1.155) | 0.144 |
|  | Model 2 | 1 | Ref (1) | 0.847 | (0.551,1.304) | 0.649 | (0.396,1.063) | 0.086 |
|  |  | 2 | Ref (1) | 0.794 | (0.547,1.151) | 0.680 | (0.444,1.039) | 0.069 |
|  |  | 3 | Ref (1) | 0.885 | (0.532,1.472) | 0.693 | (0.379,1.269) | 0.240 |
|  | Model 3 | 1 | Ref (1) | 0.859 | (0.558,1.323) | 0.650 | (0.397,1.065) | 0.088 |
|  |  | 2 | Ref (1) | 0.799 | (0.550,1.161) | 0.695 | (0.454,1.065) | 0.089 |
|  |  | 3 | Ref (1) | 0.885 | (0.532,1.470) | 0.661 | (0.360,1.213) | 0.188 |
|  | Model 4 | 1 | Ref (1) | 0.855 | (0.555,1.317) | 0.651 | (0.397,1.066) | 0.088 |
|  |  | 2 | Ref (1) | 0.810 | (0.557,1.177) | 0.751 | (0.489,1.154) | 0.176 |
|  |  | 3 | Ref (1) | 0.885 | (0.533,1.471) | 0.660 | (0.359,1.211) | 0.186 |
| Metabolic control | | | | | | | | |
|  | Model 1 | 1 | Ref (1) | 0.749 | (0.452,1.239) | **0.542** | **(0.298,0.986)** | **0.042** |
|  |  | 2 | Ref (1) | 0.729 | (0.500,1.061) | **0.496** | **(0.321,0.768)** | **0.001** |
|  |  | 3 | Ref (1) | 0.794 | (0.508,1.241) | **0.485** | **(0.280,0.842)** | **0.010** |
|  | Model 2 | 1 | Ref (1) | 0.783 | (0.471,1.300) | 0.591 | (0.322,1.083) | 0.086 |
|  |  | 2 | Ref (1) | 0.768 | (0.526,1.121) | **0.530** | **(0.341,0.824)** | **0.005** |
|  |  | 3 | Ref (1) | 0.859 | (0.546,1.350) | **0.567** | **(0.324,0.990)** | **0.049** |
|  | Model 3 | 1 | Ref (1) | 0.737 | (0.442,1.229) | 0.560 | (0.305,1.030) | 0.058 |
|  |  | 2 | Ref (1) | 0.767 | (0.525,1.121) | **0.536** | **(0.345,0.833)** | **0.005** |
|  |  | 3 | Ref (1) | 0.870 | (0.553,1.368) | 0.583 | (0.333,1.019) | 0.062 |
|  | Model 4 | 1 | Ref (1) | 0.827 | (0.495,1.383) | 0.616 | (0.334,1.136) | 0.120 |
|  |  | 2 | Ref (1) | 0.761 | (0.521,1.112) | **0.557** | **(0.358,0.867)** | **0.009** |
|  |  | 3 | Ref (1) | 0.897 | (0.569,1.414) | 0.614 | (0.350,1.079) | 0.097 |
| CVD standard PRS | | | | | | | | |
|  | Model 1 | ≤ -0.44 | Ref (1) | **0.570** | **(0.345,0.942)** | 0.720 | (0.437,1.187) | 0.198 |
|  |  | -0.44 - 0.39 | Ref (1) | 0.811 | (0.547,1.200) | **0.389** | **(0.232,0.650)** | **0.000** |
|  |  | ≥ 0.39 | Ref (1) | 0.936 | (0.631,1.390) | 0.731 | (0.464,1.152) | 0.184 |
|  | Model 2 | ≤ -0.44 | Ref (1) | 0.634 | (0.382,1.051) | 0.828 | (0.498,1.374) | 0.446 |
|  |  | -0.44 - 0.39 | Ref (1) | 0.868 | (0.584,1.289) | **0.421** | **(0.250,0.709)** | **0.001** |
|  |  | ≥ 0.39 | Ref (1) | 0.997 | (0.669,1.484) | 0.831 | (0.524,1.317) | 0.450 |
|  | Model 3 | ≤ -0.44 | Ref (1) | 0.640 | (0.385,1.064) | 0.830 | (0.499,1.378) | 0.455 |
|  |  | -0.44 - 0.39 | Ref (1) | 0.867 | (0.583,1.290) | **0.423** | **(0.251,0.712)** | **0.002** |
|  |  | ≥ 0.39 | Ref (1) | 1.011 | (0.678,1.508) | 0.837 | (0.527,1.328) | 0.471 |
|  | Model 4 | ≤ -0.44 | Ref (1) | **0.566** | **(0.333,0.963)** | 0.761 | (0.449,1.289) | 0.281 |
|  |  | -0.44 - 0.39 | Ref (1) | 0.895 | (0.600,1.333) | **0.449** | **(0.266,0.758)** | **0.004** |
|  |  | ≥ 0.39 | Ref (1) | 1.069 | (0.711,1.608) | 0.924 | (0.576,1.482) | 0.770 |
| T2DM standard PRS | | | | | | | | |
|  | Model 1 | ≤ -0.0003 | Ref (1) | 0.857 | (0.535,1.374) | 0.675 | (0.396,1.151) | 0.149 |
|  |  | -0.0003 - 0.8628 | Ref (1) | **0.624** | **(0.424,0.917)** | **0.518** | **(0.336,0.799)** | **0.002** |
|  |  | ≥ 0.8628 | Ref (1) | 0.932 | (0.612,1.419) | **0.590** | **(0.357,0.974)** | **0.043** |
|  | Model 2 | ≤ -0.0003 | Ref (1) | 0.875 | (0.545,1.404) | 0.694 | (0.405,1.190) | 0.185 |
|  |  | -0.0003 - 0.8628 | Ref (1) | 0.706 | (0.478,1.043) | **0.605** | **(0.390,0.938)** | **0.021** |
|  |  | ≥ 0.8628 | Ref (1) | 1.018 | (0.665,1.559) | 0.673 | (0.405,1.120) | 0.145 |
|  | Model 3 | ≤ -0.0003 | Ref (1) | 0.863 | (0.537,1.388) | 0.686 | (0.400,1.176) | 0.171 |
|  |  | -0.0003 - 0.8628 | Ref (1) | 0.718 | (0.485,1.062) | **0.609** | **(0.392,0.944)** | **0.023** |
|  |  | ≥ 0.8628 | Ref (1) | 1.019 | (0.665,1.562) | 0.673 | (0.404,1.121) | 0.146 |
|  | Model 4 | ≤ -0.0003 | Ref (1) | 0.834 | (0.515,1.350) | 0.652 | (0.376,1.130) | 0.127 |
|  |  | -0.0003 - 0.8628 | Ref (1) | 0.762 | (0.510,1.137) | 0.670 | (0.426,1.054) | 0.076 |
|  |  | ≥ 0.8628 | Ref (1) | 1.050 | (0.680,1.621) | 0.691 | (0.411,1.161) | 0.188 |

^a^The variables adjusted in each model were the factors mentioned above except the stratification variables.

CVD: cardiovascular disease; T2DM: Type 2 Diabetes Mellitus; PRS: Polygenic Risk Score; Ref, reference; HR: hazard ratio; CI: confidence interval.

| **Table S14.** Stratified analysis of the association between sleep patterns and Atherosclerosis of the Arteries | | | | | | | | |
| --- | --- | --- | --- | --- | --- | --- | --- | --- |
|  |  |  | Poor sleep | Intermediate sleep | | Sleep health | | P_trend_ |
|  |  |  |  | HR | 95% CI | HR | 95% CI |  |
| Age | | | | | | | |  |
|  | Model 1^a^ | ≤ 60 | Ref (1) | **0.707** | **(0.566,0.883)** | **0.605** | **(0.472,0.776)** | **0.000** |
|  |  | > 60 | Ref (1) | **0.765** | **(0.643,0.910)** | **0.711** | **(0.590,0.857)** | **0.000** |
|  | Model 2 | ≤ 60 | Ref (1) | **0.724** | **(0.579,0.905)** | **0.633** | **(0.493,0.814)** | **0.000** |
|  |  | > 60 | Ref (1) | **0.813** | **(0.683,0.969)** | **0.785** | **(0.650,0.948)** | **0.012** |
|  | Model 3 | ≤ 60 | Ref (1) | **0.718** | **(0.574,0.898)** | **0.630** | **(0.490,0.809)** | **0.000** |
|  |  | > 60 | Ref (1) | **0.817** | **(0.685,0.973)** | **0.791** | **(0.655,0.956)** | **0.015** |
|  | Model 4 | ≤ 60 | Ref (1) | **0.750** | **(0.594,0.946)** | **0.672** | **(0.519,0.871)** | **0.002** |
|  |  | > 60 | Ref (1) | 0.845 | (0.707,1.011) | **0.797** | **(0.656,0.969)** | **0.022** |
| Sex | | | | | | | |  |
|  | Model 1 | Female | Ref (1) | 0.816 | (0.631,1.055) | **0.612** | **(0.440,0.851)** | **0.003** |
|  |  | Male | Ref (1) | **0.715** | **(0.609,0.841)** | **0.690** | **(0.583,0.817)** | **0.000** |
|  | Model 2 | Female | Ref (1) | 0.875 | (0.676,1.132) | **0.674** | **(0.484,0.940)** | **0.021** |
|  |  | Male | Ref (1) | **0.757** | **(0.644,0.890)** | **0.756** | **(0.638,0.896)** | **0.001** |
|  | Model 3 | Female | Ref (1) | 0.876 | (0.677,1.135) | **0.673** | **(0.483,0.939)** | **0.021** |
|  |  | Male | Ref (1) | **0.761** | **(0.647,0.895)** | **0.760** | **(0.641,0.901)** | **0.002** |
|  | Model 4 | Female | Ref (1) | 0.912 | (0.699,1.189) | **0.681** | **(0.483,0.959)** | **0.034** |
|  |  | Male | Ref (1) | **0.801** | **(0.678,0.946)** | **0.801** | **(0.671,0.955)** | **0.014** |
| Education | | | | | | | |  |
|  | Model 1 | < High school | Ref (1) | **0.661** | **(0.518,0.844)** | **0.568** | **(0.432,0.748)** | **0.000** |
|  |  | $\geq$High school | Ref (1) | **0.781** | **(0.662,0.921)** | **0.734** | **(0.614,0.877)** | **0.001** |
|  | Model 2 | < High school | Ref (1) | **0.720** | **(0.564,0.920)** | **0.644** | **(0.487,0.851)** | **0.001** |
|  |  | $\geq$High school | Ref (1) | **0.818** | **(0.693,0.966)** | **0.791** | **(0.661,0.946)** | **0.010** |
|  | Model 3 | < High school | Ref (1) | **0.727** | **(0.569,0.929)** | **0.648** | **(0.490,0.857)** | **0.002** |
|  |  | $\geq$High school | Ref (1) | **0.819** | **(0.693,0.967)** | **0.792** | **(0.662,0.948)** | **0.010** |
|  | Model 4 | < High school | Ref (1) | **0.746** | **(0.578,0.962)** | **0.678** | **(0.507,0.905)** | **0.006** |
|  |  | $\geq$High school | Ref (1) | 0.864 | (0.729,1.025) | **0.821** | **(0.682,0.988)** | **0.036** |
| Townsend deprivation index | | | | | | | |  |
|  | Model 1 | ≤ -3.13 | Ref (1) | **0.748** | **(0.576,0.972)** | **0.716** | **(0.542,0.946)** | **0.021** |
|  |  | -3.13 - -0.58 | Ref (1) | **0.764** | **(0.602,0.970)** | **0.662** | **(0.512,0.856)** | **0.002** |
|  |  | ≥ -0.58 | Ref (1) | **0.749** | **(0.602,0.933)** | **0.728** | **(0.570,0.930)** | **0.007** |
|  | Model 2 | ≤ -3.13 | Ref (1) | **0.748** | **(0.576,0.972)** | **0.725** | **(0.548,0.960)** | **0.028** |
|  |  | -3.13 - -0.58 | Ref (1) | 0.790 | (0.622,1.004) | **0.703** | **(0.542,0.912)** | **0.007** |
|  |  | ≥ -0.58 | Ref (1) | **0.789** | **(0.633,0.983)** | **0.781** | **(0.610,0.999)** | **0.034** |
|  | Model 3 | ≤ -3.13 | Ref (1) | **0.747** | **(0.575,0.971)** | **0.725** | **(0.547,0.961)** | **0.028** |
|  |  | -3.13 - -0.58 | Ref (1) | 0.794 | (0.625,1.009) | **0.709** | **(0.547,0.920)** | **0.009** |
|  |  | ≥ -0.58 | Ref (1) | **0.786** | **(0.631,0.980)** | 0.784 | (0.612,1.004) | **0.037** |
|  | Model 4 | ≤ -3.13 | Ref (1) | 0.772 | (0.590,1.010) | **0.724** | **(0.541,0.968)** | **0.032** |
|  |  | -3.13 - -0.58 | Ref (1) | 0.830 | (0.649,1.062) | **0.740** | **(0.567,0.967)** | **0.026** |
|  |  | ≥ -0.58 | Ref (1) | 0.835 | (0.666,1.048) | 0.845 | (0.654,1.092) | 0.160 |
| Age at diagnosis of type 2 diabetes | | | | | | | |  |
|  | Model 1 | ≤ 54 | Ref (1) | **0.735** | **(0.554,0.975)** | **0.669** | **(0.493,0.908)** | **0.008** |
|  |  | 54 - 61 | Ref (1) | **0.697** | **(0.560,0.868)** | **0.535** | **(0.414,0.690)** | **0.000** |
|  |  | ≥ 62 | Ref (1) | **0.795** | **(0.635,0.995)** | 0.837 | (0.662,1.058) | 0.146 |
|  | Model 2 | ≤ 54 | Ref (1) | **0.739** | **(0.556,0.982)** | 0.743 | (0.546,1.011) | **0.046** |
|  |  | 54 - 61 | Ref (1) | **0.728** | **(0.584,0.907)** | **0.576** | **(0.445,0.746)** | **0.000** |
|  |  | ≥ 62 | Ref (1) | 0.851 | (0.679,1.067) | 0.928 | (0.732,1.177) | 0.548 |
|  | Model 3 | ≤ 54 | Ref (1) | **0.748** | **(0.562,0.995)** | 0.749 | (0.550,1.019) | 0.053 |
|  |  | 54 - 61 | Ref (1) | **0.731** | **(0.586,0.913)** | **0.580** | **(0.448,0.752)** | **0.000** |
|  |  | ≥ 62 | Ref (1) | 0.852 | (0.679,1.068) | 0.930 | (0.734,1.179) | 0.560 |
|  | Model 4 | ≤ 54 | Ref (1) | 0.794 | (0.592,1.067) | 0.802 | (0.581,1.107) | 0.157 |
|  |  | 54 - 61 | Ref (1) | **0.752** | **(0.597,0.946)** | **0.618** | **(0.473,0.806)** | **0.000** |
|  |  | ≥ 62 | Ref (1) | 0.888 | (0.706,1.117) | 0.919 | (0.721,1.172) | 0.503 |
| Severity of type 2 diabetes | | | | | | | |  |
|  | Model 1 | 1 | Ref (1) | **0.767** | **(0.624,0.943)** | **0.699** | **(0.558,0.876)** | **0.002** |
|  |  | 2 | Ref (1) | 0.806 | (0.636,1.022) | **0.745** | **(0.577,0.961)** | **0.023** |
|  |  | 3 | Ref (1) | 0.767 | (0.552,1.065) | **0.615** | **(0.423,0.895)** | **0.010** |
|  | Model 2 | 1 | Ref (1) | 0.813 | (0.660,1.001) | **0.755** | **(0.601,0.949)** | **0.015** |
|  |  | 2 | Ref (1) | 0.840 | (0.662,1.066) | 0.826 | (0.638,1.068) | 0.139 |
|  |  | 3 | Ref (1) | 0.804 | (0.576,1.122) | 0.689 | (0.470,1.010) | 0.050 |
|  | Model 3 | 1 | Ref (1) | **0.810** | **(0.658,0.998)** | **0.755** | **(0.601,0.949)** | **0.015** |
|  |  | 2 | Ref (1) | 0.859 | (0.676,1.092) | 0.846 | (0.653,1.096) | 0.199 |
|  |  | 3 | Ref (1) | 0.798 | (0.572,1.113) | **0.673** | **(0.458,0.989)** | **0.039** |
|  | Model 4 | 1 | Ref (1) | 0.819 | (0.665,1.009) | **0.753** | **(0.599,0.946)** | **0.014** |
|  |  | 2 | Ref (1) | 0.864 | (0.680,1.098) | 0.894 | (0.689,1.160) | 0.382 |
|  |  | 3 | Ref (1) | 0.804 | (0.576,1.122) | 0.682 | (0.464,1.002) | **0.046** |
| Metabolic control | | | | | | | |  |
|  | Model 1 | 1 | Ref (1) | 0.788 | (0.596,1.044) | **0.631** | **(0.459,0.868)** | **0.004** |
|  |  | 2 | Ref (1) | 0.871 | (0.699,1.086) | **0.689** | **(0.541,0.878)** | **0.003** |
|  |  | 3 | Ref (1) | **0.621** | **(0.472,0.818)** | **0.694** | **(0.521,0.926)** | **0.014** |
|  | Model 2 | 1 | Ref (1) | 0.822 | (0.620,1.091) | **0.656** | **(0.475,0.905)** | **0.010** |
|  |  | 2 | Ref (1) | 0.918 | (0.735,1.146) | **0.781** | **(0.611,0.997)** | **0.049** |
|  |  | 3 | Ref (1) | **0.675** | **(0.511,0.891)** | 0.795 | (0.593,1.066) | 0.123 |
|  | Model 3 | 1 | Ref (1) | 0.821 | (0.618,1.090) | **0.656** | **(0.475,0.906)** | **0.010** |
|  |  | 2 | Ref (1) | 0.928 | (0.743,1.159) | 0.792 | (0.620,1.012) | 0.064 |
|  |  | 3 | Ref (1) | **0.682** | **(0.516,0.902)** | 0.799 | (0.595,1.072) | 0.134 |
|  | Model 4 | 1 | Ref (1) | 0.868 | (0.653,1.153) | **0.682** | **(0.493,0.943)** | **0.021** |
|  |  | 2 | Ref (1) | 0.933 | (0.747,1.165) | 0.811 | (0.635,1.036) | 0.096 |
|  |  | 3 | Ref (1) | **0.702** | **(0.530,0.929)** | 0.828 | (0.616,1.113) | 0.209 |
| CVD standard PRS | | | | | | | |  |
|  | Model 1 | ≤ -0.46 | Ref (1) | **0.625** | **(0.467,0.836)** | **0.726** | **(0.541,0.976)** | **0.039** |
|  |  | -0.46 - 0.37 | Ref (1) | **0.764** | **(0.604,0.966)** | **0.627** | **(0.481,0.815)** | **0.000** |
|  |  | ≥ 0.37 | Ref (1) | **0.794** | **(0.642,0.982)** | **0.693** | **(0.547,0.876)** | **0.002** |
|  | Model 2 | ≤ -0.46 | Ref (1) | **0.691** | **(0.516,0.925)** | 0.804 | (0.596,1.085) | 0.158 |
|  |  | -0.46 - 0.37 | Ref (1) | 0.804 | (0.635,1.018) | **0.677** | **(0.519,0.884)** | **0.004** |
|  |  | ≥ 0.37 | Ref (1) | 0.826 | (0.667,1.023) | **0.760** | **(0.599,0.965)** | **0.021** |
|  | Model 3 | ≤ -0.46 | Ref (1) | **0.688** | **(0.514,0.923)** | 0.799 | (0.592,1.078) | 0.147 |
|  |  | -0.46 - 0.37 | Ref (1) | 0.809 | (0.639,1.025) | **0.684** | **(0.523,0.893)** | **0.005** |
|  |  | ≥ 0.37 | Ref (1) | 0.830 | (0.670,1.029) | **0.765** | **(0.603,0.971)** | **0.024** |
|  | Model 4 | ≤ -0.46 | Ref (1) | **0.709** | **(0.525,0.959)** | 0.788 | (0.578,1.075) | 0.137 |
|  |  | -0.46 - 0.37 | Ref (1) | 0.862 | (0.678,1.097) | **0.711** | **(0.542,0.933)** | **0.014** |
|  |  | ≥ 0.37 | Ref (1) | 0.847 | (0.681,1.055) | 0.801 | (0.627,1.024) | 0.069 |
| T2DM standard PRS | | | | | | | |  |
|  | Model 1 | ≤ -0.0114 | Ref (1) | **0.649** | **(0.503,0.838)** | **0.601** | **(0.456,0.793)** | **0.000** |
|  |  | -0.0114 - 0.8543 | Ref (1) | 0.801 | (0.632,1.015) | **0.668** | **(0.514,0.869)** | **0.002** |
|  |  | ≥ 0.8543 | Ref (1) | **0.737** | **(0.586,0.928)** | **0.722** | **(0.564,0.924)** | **0.008** |
|  | Model 2 | ≤ -0.0114 | Ref (1) | **0.649** | **(0.502,0.839)** | **0.640** | **(0.484,0.846)** | **0.001** |
|  |  | -0.0114 - 0.8543 | Ref (1) | 0.867 | (0.683,1.102) | **0.746** | **(0.572,0.974)** | **0.031** |
|  |  | ≥ 0.8543 | Ref (1) | 0.804 | (0.637,1.014) | 0.808 | (0.629,1.038) | 0.086 |
|  | Model 3 | ≤ -0.0114 | Ref (1) | **0.646** | **(0.500,0.835)** | **0.637** | **(0.481,0.843)** | **0.001** |
|  |  | -0.0114 - 0.8543 | Ref (1) | 0.870 | (0.684,1.106) | **0.747** | **(0.572,0.976)** | **0.032** |
|  |  | ≥ 0.8543 | Ref (1) | 0.806 | (0.639,1.017) | 0.811 | (0.631,1.042) | 0.092 |
|  | Model 4 | ≤ -0.0114 | Ref (1) | **0.656** | **(0.504,0.854)** | **0.628** | **(0.470,0.839)** | **0.001** |
|  |  | -0.0114 - 0.8543 | Ref (1) | 0.951 | (0.744,1.216) | 0.835 | (0.634,1.098) | 0.200 |
|  |  | ≥ 0.8543 | Ref (1) | 0.830 | (0.655,1.053) | 0.800 | (0.619,1.036) | 0.085 |

^a^The variables adjusted in each model were the factors mentioned above except the stratification variables.

CVD: cardiovascular disease; T2DM: Type 2 Diabetes Mellitus; PRS: Polygenic Risk Score; Ref, reference; HR: hazard ratio; CI: confidence interval.

| **Table S15.** Stratified analysis of the association between sleep patterns and Coronary Artery Disease | | | | | | | | |
| --- | --- | --- | --- | --- | --- | --- | --- | --- |
|  |  |  | Poor sleep | Intermediate sleep | | Sleep health | | P_trend_ |
|  |  |  |  | HR | 95% CI | HR | 95% CI |  |
| Age | | | | | | | | |
|  | Model 1^a^ | ≤ 60 | Ref (1) | **0.590** | **(0.457,0.762)** | **0.639** | **(0.490,0.833)** | **0.001** |
|  |  | > 60 | Ref (1) | **0.790** | **(0.645,0.966)** | **0.700** | **(0.562,0.872)** | **0.001** |
|  | Model 2 | ≤ 60 | Ref (1) | **0.598** | **(0.463,0.773)** | **0.659** | **(0.505,0.861)** | **0.001** |
|  |  | > 60 | Ref (1) | 0.819 | (0.668,1.004) | **0.746** | **(0.597,0.931)** | **0.010** |
|  | Model 3 | ≤ 60 | Ref (1) | **0.598** | **(0.463,0.773)** | **0.657** | **(0.503,0.858)** | **0.001** |
|  |  | > 60 | Ref (1) | 0.825 | (0.673,1.011) | **0.757** | **(0.606,0.946)** | **0.014** |
|  | Model 4 | ≤ 60 | Ref (1) | **0.623** | **(0.478,0.812)** | **0.666** | **(0.504,0.880)** | **0.003** |
|  |  | > 60 | Ref (1) | 0.853 | (0.693,1.050) | **0.758** | **(0.603,0.953)** | **0.018** |
| Sex | | | | | | | | |
|  | Model 1 | Female | Ref (1) | **0.734** | **(0.551,0.978)** | **0.652** | **(0.460,0.925)** | **0.009** |
|  |  | Male | Ref (1) | **0.692** | **(0.573,0.835)** | **0.682** | **(0.562,0.829)** | **0.000** |
|  | Model 2 | Female | Ref (1) | 0.788 | (0.590,1.051) | 0.711 | (0.500,1.012) | **0.041** |
|  |  | Male | Ref (1) | **0.707** | **(0.585,0.854)** | **0.715** | **(0.588,0.869)** | **0.001** |
|  | Model 3 | Female | Ref (1) | 0.787 | (0.590,1.050) | 0.715 | (0.503,1.018) | **0.044** |
|  |  | Male | Ref (1) | **0.713** | **(0.590,0.862)** | **0.723** | **(0.594,0.879)** | **0.001** |
|  | Model 4 | Female | Ref (1) | 0.789 | (0.588,1.059) | **0.682** | **(0.473,0.985)** | **0.030** |
|  |  | Male | Ref (1) | **0.750** | **(0.618,0.911)** | **0.744** | **(0.607,0.912)** | **0.005** |
| Education | | | | | | | | |
|  | Model 1 | < High school | Ref (1) | **0.650** | **(0.492,0.858)** | **0.569** | **(0.418,0.774)** | **0.000** |
|  |  | $\geq$High school | Ref (1) | **0.734** | **(0.606,0.889)** | **0.735** | **(0.600,0.900)** | **0.003** |
|  | Model 2 | < High school | Ref (1) | **0.673** | **(0.509,0.889)** | **0.597** | **(0.437,0.814)** | **0.001** |
|  |  | $\geq$High school | Ref (1) | **0.757** | **(0.624,0.917)** | **0.776** | **(0.632,0.951)** | **0.014** |
|  | Model 3 | < High school | Ref (1) | **0.684** | **(0.516,0.905)** | **0.613** | **(0.449,0.838)** | **0.002** |
|  |  | $\geq$High school | Ref (1) | **0.756** | **(0.623,0.916)** | **0.777** | **(0.633,0.953)** | **0.014** |
|  | Model 4 | < High school | Ref (1) | **0.674** | **(0.505,0.899)** | **0.572** | **(0.412,0.793)** | **0.001** |
|  |  | $\geq$High school | Ref (1) | **0.813** | **(0.667,0.990)** | 0.819 | (0.663,1.013) | 0.062 |
| Townsend deprivation index | | | | | | | | |
|  | Model 1 | ≤ -3.13 | Ref (1) | **0.729** | **(0.540,0.983)** | **0.654** | **(0.474,0.903)** | **0.011** |
|  |  | -3.13 - -0.58 | Ref (1) | 0.754 | (0.566,1.005) | 0.904 | (0.679,1.202) | 0.548 |
|  |  | ≥ -0.58 | Ref (1) | **0.681** | **(0.533,0.870)** | **0.558** | **(0.418,0.745)** | **0.000** |
|  | Model 2 | ≤ -3.13 | Ref (1) | **0.719** | **(0.533,0.970)** | **0.644** | **(0.466,0.890)** | **0.009** |
|  |  | -3.13 - -0.58 | Ref (1) | 0.785 | (0.589,1.048) | 0.961 | (0.721,1.280) | 0.843 |
|  |  | ≥ -0.58 | Ref (1) | **0.702** | **(0.549,0.899)** | **0.565** | **(0.422,0.756)** | **0.000** |
|  | Model 3 | ≤ -3.13 | Ref (1) | **0.714** | **(0.529,0.964)** | **0.644** | **(0.466,0.892)** | **0.009** |
|  |  | -3.13 - -0.58 | Ref (1) | 0.793 | (0.594,1.058) | 0.976 | (0.732,1.301) | 0.923 |
|  |  | ≥ -0.58 | Ref (1) | **0.705** | **(0.550,0.903)** | **0.572** | **(0.427,0.766)** | **0.000** |
|  | Model 4 | ≤ -3.13 | Ref (1) | 0.767 | (0.563,1.046) | **0.667** | **(0.475,0.936)** | **0.020** |
|  |  | -3.13 - -0.58 | Ref (1) | 0.846 | (0.630,1.136) | 1.008 | (0.751,1.352) | 0.920 |
|  |  | ≥ -0.58 | Ref (1) | **0.722** | **(0.561,0.930)** | **0.554** | **(0.407,0.754)** | **0.000** |
| Age at diagnosis of type 2 diabetes | | | | | | | | |
|  | Model 1 | ≤ 54 | Ref (1) | **0.663** | **(0.488,0.899)** | **0.636** | **(0.461,0.877)** | **0.004** |
|  |  | 54 - 61 | Ref (1) | **0.665** | **(0.508,0.869)** | **0.695** | **(0.523,0.924)** | **0.011** |
|  |  | ≥ 62 | Ref (1) | 0.786 | (0.609,1.015) | **0.699** | **(0.529,0.923)** | **0.011** |
|  | Model 2 | ≤ 54 | Ref (1) | **0.669** | **(0.493,0.908)** | **0.668** | **(0.483,0.924)** | **0.011** |
|  |  | 54 - 61 | Ref (1) | **0.652** | **(0.497,0.854)** | **0.725** | **(0.543,0.967)** | **0.025** |
|  |  | ≥ 62 | Ref (1) | 0.829 | (0.641,1.072) | **0.746** | **(0.564,0.988)** | **0.041** |
|  | Model 3 | ≤ 54 | Ref (1) | **0.690** | **(0.508,0.939)** | **0.683** | **(0.494,0.946)** | **0.017** |
|  |  | 54 - 61 | Ref (1) | **0.648** | **(0.494,0.850)** | **0.724** | **(0.542,0.966)** | **0.024** |
|  |  | ≥ 62 | Ref (1) | 0.827 | (0.640,1.071) | **0.753** | **(0.569,0.997)** | **0.047** |
|  | Model 4 | ≤ 54 | Ref (1) | **0.714** | **(0.519,0.982)** | 0.715 | (0.509,1.006) | **0.044** |
|  |  | 54 - 61 | Ref (1) | **0.678** | **(0.514,0.894)** | 0.748 | (0.555,1.009) | **0.049** |
|  |  | ≥ 62 | Ref (1) | 0.861 | (0.663,1.118) | **0.741** | **(0.555,0.988)** | **0.041** |
| Severity of type 2 diabetes | | | | | | | | |
|  | Model 1 | 1 | Ref (1) | **0.763** | **(0.601,0.970)** | **0.619** | **(0.474,0.808)** | **0.000** |
|  |  | 2 | Ref (1) | **0.745** | **(0.565,0.982)** | 0.756 | (0.565,1.012) | 0.059 |
|  |  | 3 | Ref (1) | 0.705 | (0.494,1.007) | 0.699 | (0.475,1.028) | 0.055 |
|  | Model 2 | 1 | Ref (1) | 0.789 | (0.620,1.004) | **0.640** | **(0.489,0.837)** | **0.001** |
|  |  | 2 | Ref (1) | **0.747** | **(0.566,0.986)** | 0.800 | (0.597,1.074) | 0.131 |
|  |  | 3 | Ref (1) | 0.733 | (0.512,1.051) | 0.770 | (0.520,1.139) | 0.152 |
|  | Model 3 | 1 | Ref (1) | 0.791 | (0.621,1.006) | **0.643** | **(0.492,0.841)** | **0.001** |
|  |  | 2 | Ref (1) | 0.757 | (0.573,1.002) | 0.819 | (0.610,1.101) | 0.178 |
|  |  | 3 | Ref (1) | 0.747 | (0.521,1.071) | 0.789 | (0.532,1.169) | 0.193 |
|  | Model 4 | 1 | Ref (1) | 0.793 | (0.623,1.009) | **0.643** | **(0.492,0.841)** | **0.001** |
|  |  | 2 | Ref (1) | **0.751** | **(0.568,0.994)** | 0.859 | (0.638,1.156) | 0.294 |
|  |  | 3 | Ref (1) | 0.753 | (0.524,1.081) | 0.794 | (0.536,1.176) | 0.206 |
| Metabolic control | | | | | | | | |
|  | Model 1 | 1 | Ref (1) | **0.574** | **(0.420,0.783)** | **0.491** | **(0.348,0.694)** | **0.000** |
|  |  | 2 | Ref (1) | 0.823 | (0.644,1.052) | **0.707** | **(0.543,0.922)** | **0.010** |
|  |  | 3 | Ref (1) | 0.812 | (0.578,1.141) | 0.755 | (0.521,1.094) | 0.141 |
|  | Model 2 | 1 | Ref (1) | **0.599** | **(0.438,0.820)** | **0.508** | **(0.358,0.721)** | **0.000** |
|  |  | 2 | Ref (1) | 0.855 | (0.668,1.094) | **0.756** | **(0.578,0.988)** | **0.039** |
|  |  | 3 | Ref (1) | 0.844 | (0.599,1.189) | 0.810 | (0.556,1.178) | 0.273 |
|  | Model 3 | 1 | Ref (1) | **0.595** | **(0.435,0.814)** | **0.505** | **(0.356,0.717)** | **0.000** |
|  |  | 2 | Ref (1) | 0.850 | (0.664,1.088) | 0.766 | (0.586,1.000) | **0.049** |
|  |  | 3 | Ref (1) | 0.868 | (0.615,1.224) | 0.830 | (0.569,1.210) | 0.335 |
|  | Model 4 | 1 | Ref (1) | **0.611** | **(0.447,0.837)** | **0.519** | **(0.365,0.738)** | **0.000** |
|  |  | 2 | Ref (1) | 0.845 | (0.660,1.082) | 0.781 | (0.598,1.020) | 0.067 |
|  |  | 3 | Ref (1) | 0.878 | (0.622,1.239) | 0.864 | (0.592,1.261) | 0.451 |
| CVD standard PRS | | | | | | | | |
|  | Model 1 | ≤ -0.46 | Ref (1) | 0.783 | (0.572,1.072) | 0.745 | (0.534,1.04) | 0.086 |
|  |  | -0.46 - 0.37 | Ref (1) | **0.690** | **(0.531,0.895)** | **0.671** | **(0.507,0.889)** | **0.004** |
|  |  | ≥ 0.37 | Ref (1) | **0.674** | **(0.520,0.874)** | **0.659** | **(0.499,0.871)** | **0.003** |
|  | Model 2 | ≤ -0.46 | Ref (1) | 0.812 | (0.592,1.113) | 0.779 | (0.557,1.091) | 0.148 |
|  |  | -0.46 - 0.37 | Ref (1) | **0.721** | **(0.555,0.938)** | **0.710** | **(0.535,0.944)** | **0.015** |
|  |  | ≥ 0.37 | Ref (1) | **0.675** | **(0.520,0.878)** | **0.696** | **(0.526,0.922)** | **0.009** |
|  | Model 3 | ≤ -0.46 | Ref (1) | 0.804 | (0.586,1.103) | 0.770 | (0.550,1.078) | 0.13 |
|  |  | -0.46 - 0.37 | Ref (1) | **0.731** | **(0.562,0.951)** | **0.728** | **(0.547,0.968)** | **0.025** |
|  |  | ≥ 0.37 | Ref (1) | **0.688** | **(0.529,0.895)** | **0.709** | **(0.535,0.940)** | **0.014** |
|  | Model 4 | ≤ -0.46 | Ref (1) | 0.796 | (0.577,1.100) | 0.729 | (0.515,1.030) | 0.074 |
|  |  | -0.46 - 0.37 | Ref (1) | 0.778 | (0.596,1.016) | 0.760 | (0.568,1.017) | 0.058 |
|  |  | ≥ 0.37 | Ref (1) | **0.724** | **(0.552,0.948)** | **0.720** | **(0.535,0.969)** | **0.025** |
| T2DM standard PRS | | | | | | | | |
|  | Model 1 | ≤ -0.0114 | Ref (1) | 0.859 | (0.639,1.154) | 0.770 | (0.558,1.062) | 0.112 |
|  |  | -0.0114 - 0.8543 | Ref (1) | **0.583** | **(0.446,0.762)** | **0.549** | **(0.411,0.734)** | **0.000** |
|  |  | ≥ 0.8543 | Ref (1) | **0.711** | **(0.544,0.930)** | **0.736** | **(0.557,0.972)** | **0.029** |
|  | Model 2 | ≤ -0.0114 | Ref (1) | 0.848 | (0.630,1.142) | 0.793 | (0.572,1.099) | 0.164 |
|  |  | -0.0114 - 0.8543 | Ref (1) | **0.629** | **(0.480,0.823)** | **0.613** | **(0.457,0.821)** | **0.001** |
|  |  | ≥ 0.8543 | Ref (1) | **0.739** | **(0.564,0.968)** | 0.767 | (0.579,1.016) | 0.060 |
|  | Model 3 | ≤ -0.0114 | Ref (1) | 0.852 | (0.633,1.148) | 0.808 | (0.583,1.121) | 0.202 |
|  |  | -0.0114 - 0.8543 | Ref (1) | **0.631** | **(0.481,0.827)** | **0.616** | **(0.459,0.826)** | **0.001** |
|  |  | ≥ 0.8543 | Ref (1) | **0.745** | **(0.568,0.976)** | 0.760 | (0.574,1.007) | 0.053 |
|  | Model 4 | ≤ -0.0114 | Ref (1) | 0.820 | (0.607,1.108) | 0.771 | (0.552,1.076) | 0.125 |
|  |  | -0.0114 - 0.8543 | Ref (1) | **0.725** | **(0.549,0.957)** | **0.665** | **(0.487,0.906)** | **0.008** |
|  |  | ≥ 0.8543 | Ref (1) | **0.733** | **(0.556,0.967)** | **0.734** | **(0.549,0.981)** | **0.034** |

^a^The variables adjusted in each model were the factors mentioned above except the stratification variables.

CVD: cardiovascular disease; T2DM: Type 2 Diabetes Mellitus; PRS: Polygenic Risk Score; Ref, reference; HR: hazard ratio; CI: confidence interval.

| **Table S16.** Stratified analysis of the association between sleep patterns and Peripheral Artery Disease | | | | | | | | |
| --- | --- | --- | --- | --- | --- | --- | --- | --- |
|  |  |  | Poor sleep | Intermediate sleep | | Sleep health | | P_trend_ |
|  |  |  |  | HR | 95% CI | HR | 95% CI |  |
| Age | | | | | | | | |
|  | Model 1^a^ | ≤ 60 | Ref (1) | 0.942 | (0.546,1.626) | 0.656 | (0.348,1.238) | 0.202 |
|  |  | > 60 | Ref (1) | 0.724 | (0.497,1.057) | **0.497** | **(0.319,0.773)** | **0.002** |
|  | Model 2 | ≤ 60 | Ref (1) | 0.982 | (0.566,1.702) | 0.735 | (0.387,1.394) | 0.362 |
|  |  | > 60 | Ref (1) | 0.782 | (0.534,1.144) | **0.558** | **(0.357,0.873)** | **0.010** |
|  | Model 3 | ≤ 60 | Ref (1) | 0.987 | (0.569,1.713) | 0.733 | (0.386,1.392) | **0.036** |
|  |  | > 60 | Ref (1) | 0.796 | (0.543,1.166) | **0.561** | **(0.359,0.877)** | **0.011** |
|  | Model 4 | ≤ 60 | Ref (1) | 1.094 | (0.613,1.952) | 0.823 | (0.418,1.620) | 0.604 |
|  |  | > 60 | Ref (1) | 0.830 | (0.565,1.218) | **0.529** | **(0.332,0.841)** | **0.007** |
| Sex | | | | | | | | |
|  | Model 1 | Female | Ref (1) | 0.599 | (0.286,1.256) | 0.438 | (0.162,1.187) | 0.070 |
|  |  | Male | Ref (1) | 0.842 | (0.597,1.188) | **0.574** | **(0.387,0.850)** | **0.006** |
|  | Model 2 | Female | Ref (1) | 0.653 | (0.310,1.375) | 0.487 | (0.179,1.327) | 0.121 |
|  |  | Male | Ref (1) | 0.903 | (0.639,1.278) | **0.641** | **(0.431,0.954)** | **0.029** |
|  | Model 3 | Female | Ref (1) | 0.675 | (0.320,1.424) | 0.507 | (0.186,1.382) | 0.145 |
|  |  | Male | Ref (1) | 0.914 | (0.645,1.294) | **0.642** | **(0.431,0.955)** | **0.030** |
|  | Model 4 | Female | Ref (1) | 0.621 | (0.285,1.354) | 0.574 | (0.208,1.583) | 0.198 |
|  |  | Male | Ref (1) | 0.991 | (0.696,1.411) | **0.631** | **(0.416,0.956)** | **0.032** |
| Education | | | | | | | | |
|  | Model 1 | < High school | Ref (1) | 1.067 | (0.637,1.790) | 0.526 | (0.273,1.014) | 0.068 |
|  |  | $\geq$High school | Ref (1) | 0.677 | (0.458,1.001) | **0.562** | **(0.363,0.870)** | **0.009** |
|  | Model 2 | < High school | Ref (1) | 1.131 | (0.672,1.904) | 0.613 | (0.315,1.190) | 0.189 |
|  |  | $\geq$High school | Ref (1) | 0.715 | (0.483,1.060) | **0.609** | **(0.393,0.945)** | **0.025** |
|  | Model 3 | < High school | Ref (1) | 1.129 | (0.670,1.903) | 0.609 | (0.313,1.183) | 0.182 |
|  |  | $\geq$High school | Ref (1) | 0.730 | (0.492,1.084) | **0.615** | **(0.396,0.955)** | **0.028** |
|  | Model 4 | < High school | Ref (1) | 1.240 | (0.720,2.134) | 0.714 | (0.360,1.416) | 0.407 |
|  |  | $\geq$High school | Ref (1) | 0.758 | (0.508,1.131) | **0.564** | **(0.355,0.894)** | **0.014** |
| Townsend deprivation index | | | | | | | | |
|  | Model 1 | ≤ -3.13 | Ref (1) | 0.781 | (0.399,1.526) | 0.786 | (0.392,1.579) | 0.521 |
|  |  | -3.13 - -0.58 | Ref (1) | 0.910 | (0.537,1.541) | **0.371** | **(0.182,0.755)** | **0.007** |
|  |  | ≥ -0.58 | Ref (1) | 0.766 | (0.477,1.23) | 0.628 | (0.363,1.087) | 0.088 |
|  | Model 2 | ≤ -3.13 | Ref (1) | 0.783 | (0.400,1.535) | 0.813 | (0.403,1.640) | 0.587 |
|  |  | -3.13 - -0.58 | Ref (1) | 0.946 | (0.557,1.606) | **0.400** | **(0.195,0.817)** | **0.015** |
|  |  | ≥ -0.58 | Ref (1) | 0.796 | (0.495,1.280) | 0.667 | (0.384,1.158) | 0.140 |
|  | Model 3 | ≤ -3.13 | Ref (1) | 0.773 | (0.393,1.519) | 0.765 | (0.379,1.546) | 0.478 |
|  |  | -3.13 - -0.58 | Ref (1) | 0.943 | (0.554,1.605) | **0.400** | **(0.195,0.821)** | **0.015** |
|  |  | ≥ -0.58 | Ref (1) | 0.809 | (0.502,1.302) | 0.681 | (0.392,1.185) | 0.164 |
|  | Model 4 | ≤ -3.13 | Ref (1) | 0.784 | (0.398,1.544) | 0.719 | (0.350,1.477) | 0.380 |
|  |  | -3.13 - -0.58 | Ref (1) | 1.131 | (0.655,1.952) | **0.479** | **(0.231,0.993)** | 0.068 |
|  |  | ≥ -0.58 | Ref (1) | 0.828 | (0.509,1.348) | 0.629 | (0.348,1.137) | 0.123 |
| Age at diagnosis of type 2 diabetes | | | | | | | | |
|  | Model 1 | ≤ 54 | Ref (1) | 0.962 | (0.457,2.022) | 0.933 | (0.431,2.020) | 0.861 |
|  |  | 54 - 61 | Ref (1) | 1.034 | (0.626,1.709) | 0.564 | (0.302,1.054) | 0.083 |
|  |  | ≥ 62 | Ref (1) | **0.569** | **(0.354,0.914)** | **0.406** | **(0.233,0.707)** | **0.001** |
|  | Model 2 | ≤ 54 | Ref (1) | 1.007 | (0.477,2.127) | 1.069 | (0.487,2.349) | 0.868 |
|  |  | 54 - 61 | Ref (1) | 1.115 | (0.672,1.851) | 0.611 | (0.325,1.149) | 0.149 |
|  |  | ≥ 62 | Ref (1) | **0.619** | **(0.384,0.997)** | **0.472** | **(0.269,0.828)** | **0.007** |
|  | Model 3 | ≤ 54 | Ref (1) | 1.024 | (0.484,2.166) | 1.101 | (0.499,2.428) | 0.813 |
|  |  | 54 - 61 | Ref (1) | 1.233 | (0.738,2.061) | 0.638 | (0.338,1.203) | 0.199 |
|  |  | ≥ 62 | Ref (1) | 0.628 | (0.390,1.011) | **0.475** | **(0.271,0.834)** | **0.007** |
|  | Model 4 | ≤ 54 | Ref (1) | 1.120 | (0.510,2.458) | 1.303 | (0.562,3.018) | 0.538 |
|  |  | 54 - 61 | Ref (1) | 1.307 | (0.776,2.204) | 0.663 | (0.346,1.273) | 0.277 |
|  |  | ≥ 62 | Ref (1) | 0.657 | (0.406,1.062) | **0.427** | **(0.237,0.768)** | **0.004** |
| Metabolic control | | | | | | | | |
|  | Model 1 | 1 | Ref (1) | 0.577 | (0.309,1.079) | **0.436** | **(0.211,0.903)** | **0.020** |
|  |  | 2 | Ref (1) | 0.915 | (0.559,1.498) | 0.722 | (0.421,1.235) | 0.234 |
|  |  | 3 | Ref (1) | 0.870 | (0.456,1.658) | **0.204** | **(0.068,0.611)** | **0.004** |
|  | Model 2 | 1 | Ref (1) | 0.585 | (0.312,1.097) | **0.453** | **(0.217,0.944)** | **0.028** |
|  |  | 2 | Ref (1) | 0.980 | (0.596,1.609) | 0.812 | (0.472,1.398) | 0.456 |
|  |  | 3 | Ref (1) | 0.983 | (0.508,1.899) | **0.254** | **(0.084,0.771)** | **0.021** |
|  | Model 3 | 1 | Ref (1) | 0.584 | (0.308,1.105) | **0.462** | **(0.221,0.966)** | **0.033** |
|  |  | 2 | Ref (1) | 1.008 | (0.612,1.659) | 0.815 | (0.473,1.404) | 0.464 |
|  |  | 3 | Ref (1) | 1.025 | (0.527,1.993) | **0.263** | **(0.086,0.801)** | **0.028** |
|  | Model 4 | 1 | Ref (1) | 0.635 | (0.335,1.200) | **0.458** | **(0.218,0.962)** | **0.034** |
|  |  | 2 | Ref (1) | 1.014 | (0.616,1.669) | 0.839 | (0.487,1.447) | 0.533 |
|  |  | 3 | Ref (1) | 1.084 | (0.553,2.123) | **0.295** | **(0.096,0.908)** | 0.054 |
| CVD standard PRS | | | | | | | | |
|  | Model 1 | ≤ -0.46 | Ref (1) | **0.442** | **(0.230,0.849)** | **0.417** | **(0.207,0.842)** | **0.010** |
|  |  | -0.46 - 0.37 | Ref (1) | 0.790 | (0.455,1.369) | **0.369** | **(0.18,0.755)** | **0.006** |
|  |  | ≥ 0.37 | Ref (1) | 1.118 | (0.685,1.823) | 0.901 | (0.522,1.554) | 0.712 |
|  | Model 2 | ≤ -0.46 | Ref (1) | **0.516** | **(0.267,0.997)** | 0.491 | (0.241,1.000) | **0.038** |
|  |  | -0.46 - 0.37 | Ref (1) | 0.806 | (0.463,1.403) | **0.374** | **(0.182,0.771)** | **0.008** |
|  |  | ≥ 0.37 | Ref (1) | 1.199 | (0.732,1.963) | 1.054 | (0.606,1.835) | 0.835 |
|  | Model 3 | ≤ -0.46 | Ref (1) | 0.533 | (0.275,1.033) | 0.505 | (0.248,1.031) | **0.048** |
|  |  | -0.46 - 0.37 | Ref (1) | 0.823 | (0.472,1.435) | **0.374** | **(0.181,0.773)** | **0.008** |
|  |  | ≥ 0.37 | Ref (1) | 1.253 | (0.764,2.057) | 1.053 | (0.605,1.832) | 0.838 |
|  | Model 4 | ≤ -0.46 | Ref (1) | 0.554 | (0.284,1.084) | **0.409** | **(0.190,0.883)** | **0.017** |
|  |  | -0.46 - 0.37 | Ref (1) | 0.849 | (0.484,1.488) | **0.399** | **(0.193,0.826)** | **0.014** |
|  |  | ≥ 0.37 | Ref (1) | 1.249 | (0.757,2.061) | 1.074 | (0.605,1.907) | 0.775 |
| T2DM standard PRS | | | | | | | | |
|  | Model 1 | ≤ -0.0114 | Ref (1) | 0.756 | (0.423,1.349) | **0.369** | **(0.174,0.781)** | **0.009** |
|  |  | -0.0114 - 0.8543 | Ref (1) | 0.965 | (0.558,1.668) | 0.567 | (0.293,1.095) | 0.094 |
|  |  | ≥ 0.8543 | Ref (1) | 0.671 | (0.398,1.129) | 0.699 | (0.405,1.205) | 0.187 |
|  | Model 2 | ≤ -0.0114 | Ref (1) | 0.804 | (0.450,1.440) | **0.413** | **(0.194,0.880)** | **0.023** |
|  |  | -0.0114 - 0.8543 | Ref (1) | 1.052 | (0.606,1.827) | 0.635 | (0.326,1.234) | 0.193 |
|  |  | ≥ 0.8543 | Ref (1) | 0.768 | (0.453,1.301) | 0.835 | (0.480,1.454) | 0.502 |
|  | Model 3 | ≤ -0.0114 | Ref (1) | 0.792 | (0.441,1.421) | **0.405** | **(0.189,0.865)** | **0.020** |
|  |  | -0.0114 - 0.8543 | Ref (1) | 1.108 | (0.635,1.934) | 0.642 | (0.330,1.249) | 0.206 |
|  |  | ≥ 0.8543 | Ref (1) | 0.779 | (0.459,1.323) | 0.849 | (0.487,1.478) | 0.540 |
|  | Model 4 | ≤ -0.0114 | Ref (1) | 0.798 | (0.439,1.450) | **0.329** | **(0.148,0.734)** | **0.007** |
|  |  | -0.0114 - 0.8543 | Ref (1) | 1.297 | (0.731,2.299) | 0.820 | (0.414,1.625) | 0.623 |
|  |  | ≥ 0.8543 | Ref (1) | 0.850 | (0.497,1.452) | 0.824 | (0.460,1.476) | 0.500 |

^a^The variables adjusted in each model were the factors mentioned above except the stratification variables.

CVD: cardiovascular disease; T2DM: Type 2 Diabetes Mellitus; PRS: Polygenic Risk Score; Ref, reference; HR: hazard ratio; CI: confidence interval.

| **Table S17.** Stratified analysis of the association between sleep patterns and Heart Failure | | | | | | | | |
| --- | --- | --- | --- | --- | --- | --- | --- | --- |
|  |  |  | Poor sleep | Intermediate sleep | | Sleep health | | P_trend_ |
|  |  |  |  | HR | 95% CI | HR | 95% CI |  |
| Age | | | | | | | | |
|  | Model 1^a^ | ≤ 60 | Ref (1) | **0.447** | **(0.277,0.722)** | **0.543** | **(0.335,0.878)** | **0.007** |
|  |  | > 60 | Ref (1) | **0.725** | **(0.534,0.985)** | **0.594** | **(0.421,0.839)** | **0.003** |
|  | Model 2 | ≤ 60 | Ref (1) | **0.477** | **(0.294,0.773)** | **0.613** | **(0.376,0.997)** | **0.026** |
|  |  | > 60 | Ref (1) | 0.753 | (0.553,1.026) | **0.626** | **(0.442,0.887)** | **0.008** |
|  | Model 3 | ≤ 60 | Ref (1) | **0.473** | **(0.292,0.768)** | **0.613** | **(0.376,0.998)** | **0.026** |
|  |  | > 60 | Ref (1) | 0.760 | (0.558,1.036) | **0.629** | **(0.444,0.891)** | **0.008** |
|  | Model 4 | ≤ 60 | Ref (1) | **0.530** | **(0.324,0.869)** | 0.657 | (0.394,1.096) | 0.067 |
|  |  | > 60 | Ref (1) | 0.789 | (0.576,1.082) | **0.632** | **(0.441,0.904)** | **0.011** |
| Sex | | | | | | | | |
|  | Model 1 | Female | Ref (1) | **0.442** | **(0.264,0.741)** | **0.536** | **(0.297,0.967)** | **0.009** |
|  |  | Male | Ref (1) | **0.708** | **(0.526,0.954)** | **0.603** | **(0.437,0.832)** | **0.002** |
|  | Model 2 | Female | Ref (1) | **0.451** | **(0.268,0.761)** | 0.610 | (0.336,1.105) | **0.026** |
|  |  | Male | Ref (1) | 0.747 | (0.553,1.008) | **0.649** | **(0.470,0.898)** | **0.009** |
|  | Model 3 | Female | Ref (1) | **0.457** | **(0.271,0.771)** | 0.612 | (0.337,1.111) | **0.028** |
|  |  | Male | Ref (1) | 0.748 | (0.554,1.010) | **0.653** | **(0.472,0.904)** | **0.010** |
|  | Model 4 | Female | Ref (1) | **0.489** | **(0.287,0.832)** | 0.664 | (0.357,1.235) | 0.063 |
|  |  | Male | Ref (1) | 0.790 | (0.582,1.072) | **0.667** | **(0.477,0.933)** | **0.017** |
| Education | | | | | | | | |
|  | Model 1 | < High school | Ref (1) | **0.605** | **(0.390,0.938)** | **0.618** | **(0.388,0.984)** | **0.034** |
|  |  | $\geq$High school | Ref (1) | **0.651** | **(0.475,0.892)** | **0.562** | **(0.396,0.799)** | **0.001** |
|  | Model 2 | < High school | Ref (1) | **0.622** | **(0.400,0.969)** | 0.646 | (0.402,1.036) | 0.057 |
|  |  | $\geq$High school | Ref (1) | **0.701** | **(0.510,0.962)** | **0.618** | **(0.434,0.880)** | **0.006** |
|  | Model 3 | < High school | Ref (1) | **0.627** | **(0.403,0.976)** | 0.655 | (0.408,1.053) | 0.065 |
|  |  | $\geq$High school | Ref (1) | **0.700** | **(0.509,0.961)** | **0.617** | **(0.433,0.879)** | **0.006** |
|  | Model 4 | < High school | Ref (1) | 0.665 | (0.422,1.045) | 0.654 | (0.398,1.075) | 0.080 |
|  |  | $\geq$High school | Ref (1) | 0.755 | (0.546,1.043) | **0.643** | **(0.446,0.928)** | **0.016** |
| Townsend deprivation index | | | | | | | | |
|  | Model 1 | ≤ -3.13 | Ref (1) | 0.717 | (0.429,1.197) | 0.768 | (0.451,1.309) | 0.355 |
|  |  | -3.13 - -0.58 | Ref (1) | 0.639 | (0.406,1.006) | **0.575** | **(0.352,0.940)** | **0.024** |
|  |  | ≥ -0.58 | Ref (1) | **0.616** | **(0.415,0.913)** | **0.517** | **(0.326,0.819)** | **0.002** |
|  | Model 2 | ≤ -3.13 | Ref (1) | 0.720 | (0.430,1.204) | 0.778 | (0.456,1.329) | 0.382 |
|  |  | -3.13 - -0.58 | Ref (1) | 0.645 | (0.409,1.016) | **0.604** | **(0.369,0.990)** | **0.039** |
|  |  | ≥ -0.58 | Ref (1) | **0.658** | **(0.443,0.977)** | **0.559** | **(0.351,0.890)** | **0.008** |
|  | Model 3 | ≤ -3.13 | Ref (1) | 0.721 | (0.431,1.207) | 0.775 | (0.454,1.323) | 0.372 |
|  |  | -3.13 - -0.58 | Ref (1) | 0.644 | (0.408,1.015) | **0.601** | **(0.366,0.985)** | **0.038** |
|  |  | ≥ -0.58 | Ref (1) | **0.664** | **(0.446,0.988)** | **0.572** | **(0.358,0.913)** | **0.011** |
|  | Model 4 | ≤ -3.13 | Ref (1) | 0.772 | (0.457,1.303) | 0.769 | (0.440,1.344) | 0.369 |
|  |  | -3.13 - -0.58 | Ref (1) | 0.697 | (0.436,1.112) | 0.648 | (0.389,1.078) | 0.086 |
|  |  | ≥ -0.58 | Ref (1) | 0.716 | (0.478,1.072) | **0.599** | **(0.368,0.975)** | **0.028** |
| Age at diagnosis of type 2 diabetes | | | | | | | | |
|  | Model 1 | ≤ 54 | Ref (1) | **0.359** | **(0.192,0.671)** | 0.781 | (0.469,1.302) | 0.300 |
|  |  | 54 - 61 | Ref (1) | **0.572** | **(0.371,0.882)** | **0.527** | **(0.325,0.855)** | **0.006** |
|  |  | ≥ 62 | Ref (1) | 0.870 | (0.593,1.277) | **0.520** | **(0.325,0.832)** | **0.007** |
|  | Model 2 | ≤ 54 | Ref (1) | **0.385** | **(0.205,0.723)** | 0.940 | (0.556,1.589) | 0.703 |
|  |  | 54 - 61 | Ref (1) | **0.603** | **(0.390,0.932)** | **0.590** | **(0.362,0.964)** | **0.023** |
|  |  | ≥ 62 | Ref (1) | 0.896 | (0.609,1.318) | **0.528** | **(0.329,0.847)** | **0.009** |
|  | Model 3 | ≤ 54 | Ref (1) | **0.395** | **(0.210,0.744)** | 0.958 | (0.566,1.623) | 0.767 |
|  |  | 54 - 61 | Ref (1) | **0.595** | **(0.385,0.920)** | **0.585** | **(0.358,0.955)** | **0.021** |
|  |  | ≥ 62 | Ref (1) | 0.917 | (0.623,1.352) | **0.535** | **(0.333,0.860)** | **0.011** |
|  | Model 4 | ≤ 54 | Ref (1) | **0.422** | **(0.222,0.803)** | 1.020 | (0.588,1.769) | 0.932 |
|  |  | 54 - 61 | Ref (1) | **0.614** | **(0.393,0.960)** | **0.598** | **(0.360,0.992)** | **0.031** |
|  |  | ≥ 62 | Ref (1) | 0.969 | (0.653,1.436) | **0.541** | **(0.333,0.879)** | **0.015** |
| Severity of type 2 diabetes | | | | | | | | |
|  | Model 1 | 1 | Ref (1) | 0.717 | (0.459,1.122) | 0.744 | (0.464,1.194) | 0.216 |
|  |  | 2 | Ref (1) | 0.768 | (0.504,1.169) | **0.573** | **(0.353,0.929)** | **0.023** |
|  |  | 3 | Ref (1) | **0.529** | **(0.318,0.877)** | **0.445** | **(0.249,0.792)** | **0.003** |
|  | Model 2 | 1 | Ref (1) | 0.759 | (0.484,1.193) | 0.799 | (0.495,1.291) | 0.352 |
|  |  | 2 | Ref (1) | 0.789 | (0.517,1.205) | 0.629 | (0.386,1.026) | 0.060 |
|  |  | 3 | Ref (1) | **0.552** | **(0.332,0.918)** | **0.494** | **(0.275,0.886)** | **0.010** |
|  | Model 3 | 1 | Ref (1) | 0.769 | (0.489,1.208) | 0.803 | (0.497,1.298) | 0.364 |
|  |  | 2 | Ref (1) | 0.801 | (0.524,1.226) | 0.647 | (0.396,1.057) | 0.079 |
|  |  | 3 | Ref (1) | **0.544** | **(0.327,0.905)** | **0.486** | **(0.270,0.876)** | **0.009** |
|  | Model 4 | 1 | Ref (1) | 0.788 | (0.501,1.240) | 0.795 | (0.491,1.286) | 0.344 |
|  |  | 2 | Ref (1) | 0.804 | (0.525,1.230) | 0.669 | (0.409,1.094) | 0.104 |
|  |  | 3 | Ref (1) | **0.537** | **(0.322,0.894)** | **0.488** | **(0.271,0.879)** | **0.009** |
| Metabolic control | | | | | | | | |
|  | Model 1 | 1 | Ref (1) | 0.668 | (0.409,1.092) | **0.413** | **(0.225,0.758)** | **0.003** |
|  |  | 2 | Ref (1) | 0.779 | (0.519,1.170) | 0.675 | (0.434,1.048) | 0.077 |
|  |  | 3 | Ref (1) | **0.58** | **(0.341,0.987)** | 0.599 | (0.340,1.053) | 0.069 |
|  | Model 2 | 1 | Ref (1) | 0.736 | (0.449,1.207) | **0.473** | **(0.256,0.873)** | **0.015** |
|  |  | 2 | Ref (1) | 0.825 | (0.547,1.243) | 0.729 | (0.466,1.140) | 0.161 |
|  |  | 3 | Ref (1) | 0.600 | (0.352,1.024) | 0.619 | (0.351,1.094) | 0.091 |
|  | Model 3 | 1 | Ref (1) | 0.725 | (0.442,1.189) | **0.467** | **(0.253,0.863)** | **0.013** |
|  |  | 2 | Ref (1) | 0.815 | (0.539,1.230) | 0.732 | (0.468,1.145) | 0.166 |
|  |  | 3 | Ref (1) | 0.601 | (0.352,1.026) | 0.642 | (0.363,1.135) | 0.116 |
|  | Model 4 | 1 | Ref (1) | 0.775 | (0.472,1.270) | **0.509** | **(0.274,0.946)** | **0.031** |
|  |  | 2 | Ref (1) | 0.796 | (0.527,1.203) | 0.729 | (0.466,1.142) | 0.161 |
|  |  | 3 | Ref (1) | 0.628 | (0.367,1.074) | 0.693 | (0.389,1.233) | 0.190 |
| CVD standard PRS | | | | | | | | |
|  | Model 1 | ≤ -0.46 | Ref (1) | **0.489** | **(0.270,0.885)** | 0.639 | (0.358,1.140) | 0.129 |
|  |  | -0.46 - 0.37 | Ref (1) | 0.744 | (0.501,1.104) | **0.518** | **(0.326,0.823)** | **0.005** |
|  |  | ≥ 0.37 | Ref (1) | **0.623** | **(0.410,0.948)** | **0.600** | **(0.379,0.950)** | **0.021** |
|  | Model 2 | ≤ -0.46 | Ref (1) | 0.566 | (0.310,1.032) | 0.737 | (0.408,1.330) | 0.303 |
|  |  | -0.46 - 0.37 | Ref (1) | 0.776 | (0.522,1.153) | **0.550** | **(0.345,0.878)** | **0.012** |
|  |  | ≥ 0.37 | Ref (1) | **0.631** | **(0.414,0.963)** | 0.645 | (0.405,1.026) | **0.045** |
|  | Model 3 | ≤ -0.46 | Ref (1) | 0.574 | (0.314,1.048) | 0.746 | (0.413,1.349) | 0.324 |
|  |  | -0.46 - 0.37 | Ref (1) | 0.784 | (0.527,1.165) | **0.554** | **(0.347,0.885)** | **0.013** |
|  |  | ≥ 0.37 | Ref (1) | **0.634** | **(0.415,0.968)** | 0.650 | (0.408,1.036) | 0.050 |
|  | Model 4 | ≤ -0.46 | Ref (1) | 0.625 | (0.338,1.153) | 0.701 | (0.377,1.302) | 0.252 |
|  |  | -0.46 - 0.37 | Ref (1) | 0.845 | (0.565,1.264) | **0.596** | **(0.372,0.956)** | **0.033** |
|  |  | ≥ 0.37 | Ref (1) | 0.655 | (0.426,1.007) | 0.683 | (0.420,1.111) | 0.090 |
| T2DM standard PRS | | | | | | | | |
|  | Model 1 | ≤ -0.0114 | Ref (1) | 0.840 | (0.515,1.370) | 0.596 | (0.337,1.055) | 0.076 |
|  |  | -0.0114 - 0.8543 | Ref (1) | **0.510** | **(0.334,0.777)** | **0.588** | **(0.381,0.908)** | **0.012** |
|  |  | ≥ 0.8543 | Ref (1) | 0.657 | (0.421,1.024) | **0.527** | **(0.322,0.865)** | **0.009** |
|  | Model 2 | ≤ -0.0114 | Ref (1) | 0.856 | (0.524,1.398) | 0.624 | (0.351,1.109) | 0.109 |
|  |  | -0.0114 - 0.8543 | Ref (1) | **0.571** | **(0.373,0.874)** | 0.670 | (0.431,1.041) | 0.056 |
|  |  | ≥ 0.8543 | Ref (1) | 0.704 | (0.449,1.104) | **0.564** | **(0.341,0.934)** | **0.023** |
|  | Model 3 | ≤ -0.0114 | Ref (1) | 0.886 | (0.541,1.450) | 0.626 | (0.352,1.115) | 0.115 |
|  |  | -0.0114 - 0.8543 | Ref (1) | **0.576** | **(0.375,0.884)** | 0.680 | (0.437,1.057) | 0.066 |
|  |  | ≥ 0.8543 | Ref (1) | 0.706 | (0.450,1.106) | **0.567** | **(0.343,0.938)** | **0.024** |
|  | Model 4 | ≤ -0.0114 | Ref (1) | 0.856 | (0.521,1.407) | **0.553** | **(0.306,0.999)** | 0.051 |
|  |  | -0.0114 - 0.8543 | Ref (1) | 0.674 | (0.434,1.045) | 0.822 | (0.518,1.303) | 0.346 |
|  |  | ≥ 0.8543 | Ref (1) | 0.710 | (0.450,1.120) | **0.552** | **(0.328,0.929)** | **0.022** |

^a^The variables adjusted in each model were the factors mentioned above except the stratification variables.

CVD: cardiovascular disease; T2DM: Type 2 Diabetes Mellitus; PRS: Polygenic Risk Score; Ref, reference; HR: hazard ratio; CI: confidence interval.
